# First‐Generation Bispidine Chelators for ^213^Bi^III^ Radiopharmaceutical Applications

**DOI:** 10.1002/cmdc.202000361

**Published:** 2020-07-02

**Authors:** Frank Bruchertseifer, Peter Comba, Bodo Martin, Alfred Morgenstern, Johannes Notni, Miriam Starke, Hubert Wadepohl

**Affiliations:** ^1^ European Commission, Joint Research Centre />Directorate for Nuclear Safety and Security Hermann-von-Helmholtz-Platz 1 76344 Eggenstein-Leopoldshafen Germany; ^2^ Universität Heidelberg, Anorganisch-Chemisches Institut Neuenheimer Feld 270 69120 Heidelberg Germany; ^3^ Universität Heidelberg Interdisciplinary Center for Scientific Computing In Neuenheimer Feld 205 69120 Heidelberg Germany; ^4^ Technische Universität München Institut für Pathologie und Pathologische Anatomie Trogerstr. 18 81675 Munich Germany

## Abstract

Hepta‐ and octadentate bispidines (3,7‐diazabicyclo[3.3.1]nonane, diaza‐adamantane) with acetate, methyl‐pyridine, and methyl‐picolinate pendant groups at the amine donors of the bispidine platform have been prepared and used to investigate Bi^III^ coordination chemistry. Crystal structure and solution spectroscopic data (NMR spectroscopy and mass spectrometry) confirm that the rigid and relatively large bispidine cavity with an axially distorted geometry is well suited for Bi^III^ and in all cases forms nine‐coordinate complexes; this is supported by an established hole size and shape analysis. It follows that nonadentate bispidines probably will be more suited as bifunctional chelators for ^213^Bi^III^‐based radiopharmaceuticals. However, two isomeric picolinate‐/acetate‐based heptadentate ligands already show very efficient complexation kinetics with ^213^Bi^III^ at ambient temperature and kinetic stability that is comparable with the standard ligands used in this field. The experimentally determined hydrophilicities (log *D*_7.4_ values) show that the Bi^III^ complexes reported are relatively hydrophilic and well suited for medicinal applications. We also present a very efficient and relatively accurate method to compute charge distributions and hydrophilicities, and this will help to further optimize the systems reported here.

## Graphical abstract

**Encapsulating the potential of bismuth**: Four first‐generation hepta‐ and octadentate bispidines have been prepared for efficient radiolabeling with ^213^Bi^III^, and their ability to form thermodynamically stable and kinetically inert ^213^Bi^III^ complexes for targeted radiotherapy. With three of the ligands, radiolabeling is significantly faster than with the “gold standard” DOTA.

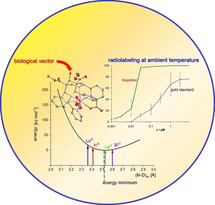

## Introduction

Interestingly, only a few years after von Hevesy's establishment of the radiotracer principle,^1^ one of the early medicinal applications and the first clinical studies with radiotracers were using bismuth‐214.^2^ Stable chelation of bismuth(III) radioisotopes is also of importance for actinium‐225 radiotherapy‐actinium‐225 is a promising nuclide for targeted α‐therapy with daughter isotopes bismuth‐211 and ‐213. Bismuth‐213 with a half‐life of 46 min may be obtained from ^225^Ac/^213^Bi generators.^3^ As Bi^III^ radiochemistry has not yet been widely established, that is, only few tailor‐made Bi^III^ chelators have been reported,^4^, ^5^, ^6^, ^7^, ^8^, ^9^, ^10^, ^11^, ^12^ there is interest in further work in this area.

As it is a borderline metal ion in terms of the hard‐soft acid‐base principle (HSAB),^13^ ligands for stable Bi^III^ complexes generally feature a combination of amine and oxygen (often carboxylate) donors, and coordination numbers from five to ten have been reported. For six‐coordinate complexes, the ionic radius is 1.03 Å, for eight‐coordinate it is 1.18 Å, with Bi^III^‐N and Bi^III^‐O distances of complexes with polyaminocarboxylate ligands around 2.40–2.65 Å and 2.25–2.60 Å, respectively.^8^, ^9^, ^14^, ^15^, ^16^

Bispidine ligands are an attractive platform in medicinal chemistry, and general applications in broad areas of coordination chemistry as well as their use in radiopharmaceutical applications have been reviewed extensively.^17^, ^18^, ^19^, ^20^ The advantages of the very rigid bispidine scaffold are: i) Ligands with a wide range of coordination numbers (4 to 8) and donor sets (mainly N and O) are accessible by relatively simple synthetic routes. 4‐ to 8‐dentate bispidines with a variety of O and N donors have been reported, and others are possible and will be developed.^18^, ^19^, ^21^ ii) There are relatively simple protocols for the preparation of bioconjugates. Various linkers have been described, and ^64^Cu^II^ tracers with bispidines conjugated to peptides or antibodies have been reported.^22^, ^23^, ^24^, ^25^ iii) There are specific bispidine ligands for a range of radiometal ions, combining high complex stability and inertness with relatively fast complexation kinetics.^18^, ^21^, ^26^, ^27^ This is due to the rigid diaza‐adamantyl backbone with two highly preorganized tertiary amines and two pendant pyridine donors as well as the possibility to attach rigid multidentate pendant groups such as picolinic acids at the tertiary amines to fully encapsulate specific metal ions in their preferred coordination geometry.^18^, ^28^

Here, we present the synthesis of three new heptadentate bispidines, together with their fully characterized Bi^III^ compounds and radiolabeling experiments with ^213^Bi^III^ of two of the heptadentate and one previously known octadentate bispidine. The structural data together with the radiolabeling efficiencies and transchelation challenge experiments, in comparison with known ligand systems, in particular with the current gold standards CHX‐A′′‐DTPA (DTPA=sodium diethylenetriamine pentaacetate) and 1,4,7,10‐tetraazacyclododecane‐1,4,7,10‐tetraacetic acid (DOTA), are used to assess the properties of these first generation bispidine bifunctional chelators towards applications for ^213^Bi^III^ targeted alpha‐therapy.


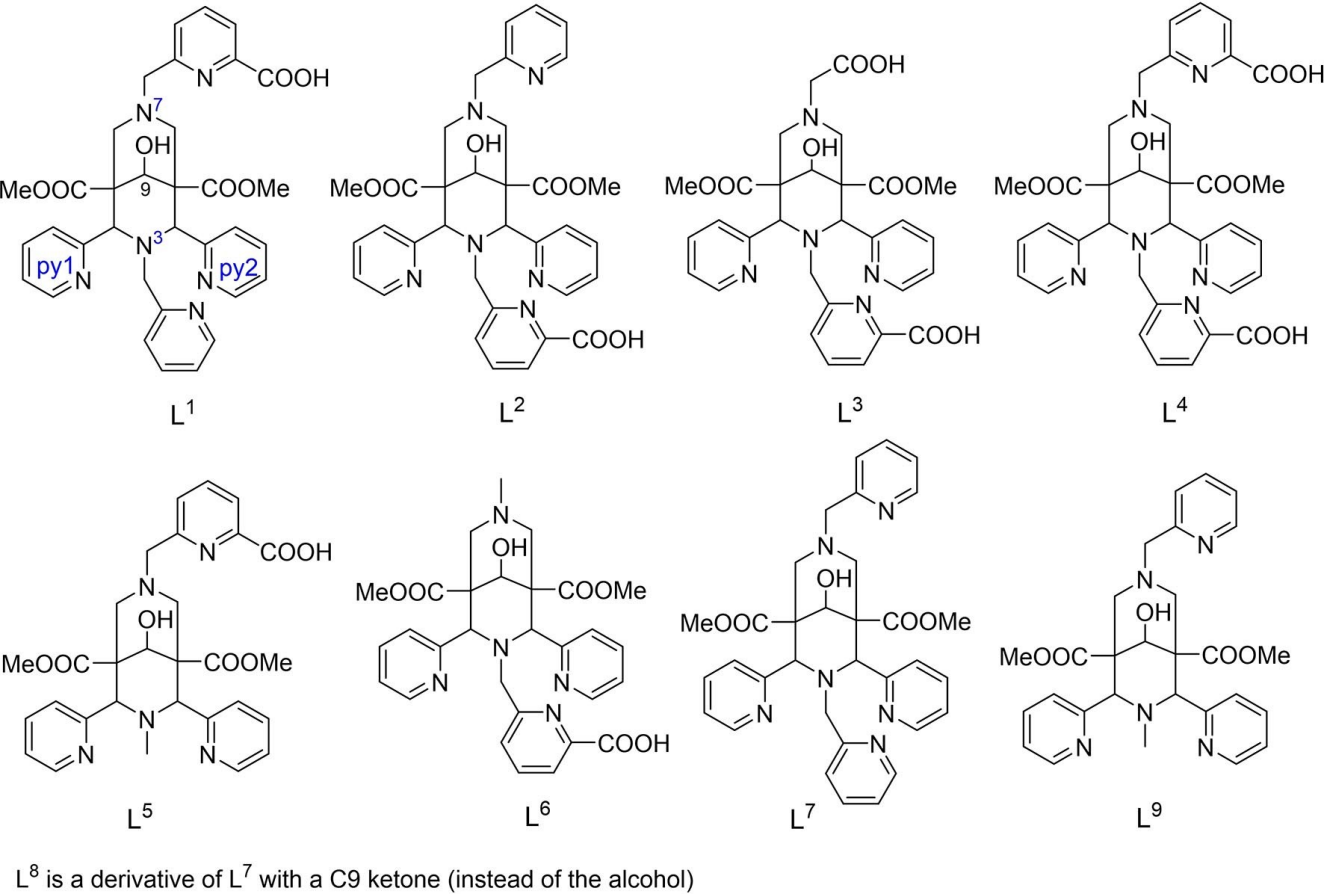


## Results and Discussion

### Ligand synthesis

**Scheme 1:**
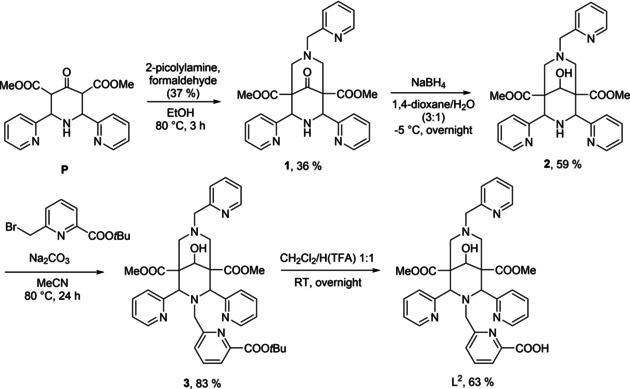
Synthesis of L^2^.

The new ligands L^1^, L^2^ and L^3^ were prepared with a methodology similar to that used for L^4^,^21^ for L^2^ this is shown in Scheme 1 (corresponding schemes for all ligands are given as Supporting Information). For L^1^ and L^2^, the bispidine platform was built up as pentadentate ligands with three pendant pyridine groups and a secondary amine (N7 for L^1^, N3 for L^2^), which was then alkylated with a picolinic acid precursor. For L^3^ and L^4^, both pendant donors (picolinic acid and acetate) had to be introduced to N3 and/or N7 by alkylation, that is, the corresponding bispidines with secondary amines had first to be prepared. For the symmetrical octadentate bispidine L^4^ this could be done in one step,^21^ while for L^3^, an additional protection/deprotection step was necessary. The overall yields of the five‐ or six‐step procedures are acceptable to good, and all four ligands were isolated as white powders and fully characterized after recrystallization. Crystal structures of the metal‐free ligands and some intermediates have been deposited (see the Supporting Information) or published.^21^

### Bispidine–bismuth(III) complexes.

**Figure 1:**
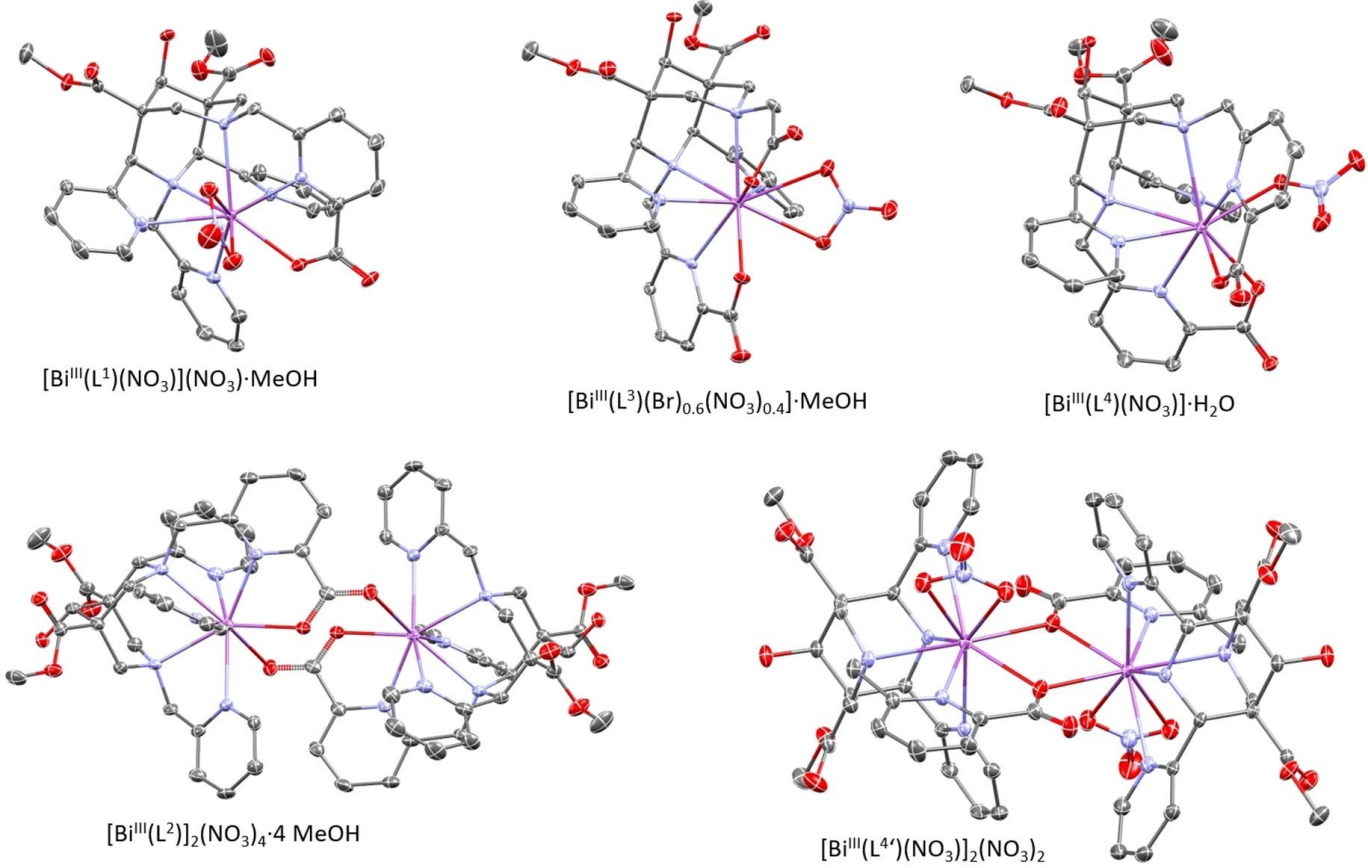
Crystal structures of [Bi^III^(L^1^)(NO_3_)](NO_3_)⋅MeOH, [Bi^III^(L^3^)(Br)_0.6_(NO_3_)_0.4_]⋅MeOH (data for the coordinated nitrate are shown), [Bi^III^(L^4^)(NO_3_)]⋅H_2_O, [Bi^III^(L^4^‘)(NO_3_)]_2_(NO_3_)_2_, and [Bi^III^(L^2^)]_2_(NO_3_)_4_⋅4 MeOH. H atoms, counter ions and solvent molecules are omitted for clarity. C: gray, N: blue, O: red, Bi: violet.

The crystalline Bi^III^ complexes were obtained in excellent yields from stoichiometric mixtures of Bi(NO_3_)_3_⋅5 H_2_O and the ligands in methanol (ca. 10 mM solutions, stirring at ambient temperature overnight). The complexes were isolated by crystallization at ambient temperature after slow evaporation of the solvent and recrystallized from MeOH. Care was taken to keep the solutions at approximately neutral pH (>4) since acidic solutions, often used to dissolve the Bi^III^ salt, might lead to partial hydrolysis of the ligands. ^1^H NMR and ^13^C NMR spectra of the complexes are given in the Supporting Information, and single crystals were subjected to X‐ray structural analyses (see below). The problem of ligand hydrolysis at low pH is demonstrated with the isolation of [Bi(L^4^’)(NO_3_)]_2_(NO_3_)_2_ with one of the two (aminomethyl)picolinic acid pendant groups hydrolyzed to the heptadentate derivative with a secondary amine at N3 (see the Experimental Section and Supporting Information for the experimental and X‐ray crystal structural details). Plots of the crystal structures of the four relevant Bi^III^ complexes are shown in Figure 1, and selected bond distances and angles are given in Table 1.

**Table 1:** Selected bond distances and angles of the X‐ray single crystal structures of [Bi^III^(L^1^)(NO_3_)](NO_3_)⋅MeOH, [Bi^III^(L^3^)(Br)_0.6_(NO_3_)_0.4_]⋅MeOH,^a)^ [Bi^III^(L^4^)(NO_3_)]⋅H_2_O, [Bi^III^(L^4^‘)(NO_3_)]_2_(NO_3_)_2_, and [Bi^III^(L^2^)]_2_(NO_3_)_4_⋅4 MeOH (see Figure 1 and Chart 1 for structural plots and nomenclature).

Ligand	L^1^	L^3 [a]^	L^4^	L^4^’	L^2^
	N_6_O_1_	N_5_O_2_	N_6_O_2_	N_5_O_1_	N_6_O_1_
CN	9	9	9	9	8
Distance [Å]					
Bi−N3	2.612(3)	2.663(2)	2.781(3)	2.512(3)	2.576(4)
Bi−N7	2.637(3)	2.563(2)	2.760(3)	2.553(3)	2.527(4)
Bi‐N^py1^	2.638(3)	2.456(3)	2.754(3)	2.580(3)	2.631(4)
Bi‐N^py2^	2.520(3)	2.720(2)	2.666(3)	2.666(3)	2.438(4)
Bi−D(N3)(N)	2.552(3)	2.457(2)	2.560(3)		2.433(4)
Bi−D(N3)(O)		2.393(2)	2.405(2)		2.451(3)
Bi−D(N7)(N)	2.480(3)		2.536(3)	2.428(3)	2.570(4)
Bi−D(N7)(O)	2.406(2)	2.361(2)	2.328(2)	2.499(3)	
Bi‐ONO_2_	2.704(3)	2.724(6)	2.538(2)	2.665(3)	
Bi‐ONO_2_	2.661(3)	2.728(5)		2.680(3)	
Bi‐O^pa2^				2.584(3)	2.570(3)
**Angle [°]**					
N3‐Bi−N7	70.28(8)	71.08(7)	67.53(7)	69.90(9)	73.37(11)
N3‐Bi‐N^py1^	61.72(8)	66.17(7)	60.05(8)	65.57(9)	64.56(12)
N3‐Bi‐N^py2^	67.56(8)	61.25(7)	63.41(8)	64.11(9)	66.44(12)
N3‐Bi−D(N3)(N)	68.94(8)	67.99(7)	65.09(8)		69.29(12)
N3‐Bi−D(N3)(O)		130.47(7)	127.42(8)		132.33(11)
N7‐Bi‐N^py1^	90.57(8)	83.89(8)	85.76(8)	81.57(10)	88.29(12)
N7‐Bi‐N^py2^	81.99(8)	88.37(7)	79.95(9)	88.68(10)	88.68(12)
N3‐Bi−D(N7)(N)	129.85(8)		118.36(8)	129.82(10)	127.24(11)
N3‐Bi−D(N7)(O)	137.74(8)	125.73(7)	128.21(8)	133.79(8)	
N^py1^‐Bi‐N^py2^	128.15(9)	126.46(8)	122.93(8)	129.03(9)	129.61(12)

[a] The data for the coordinated nitrate are shown.

**Figure 2:**
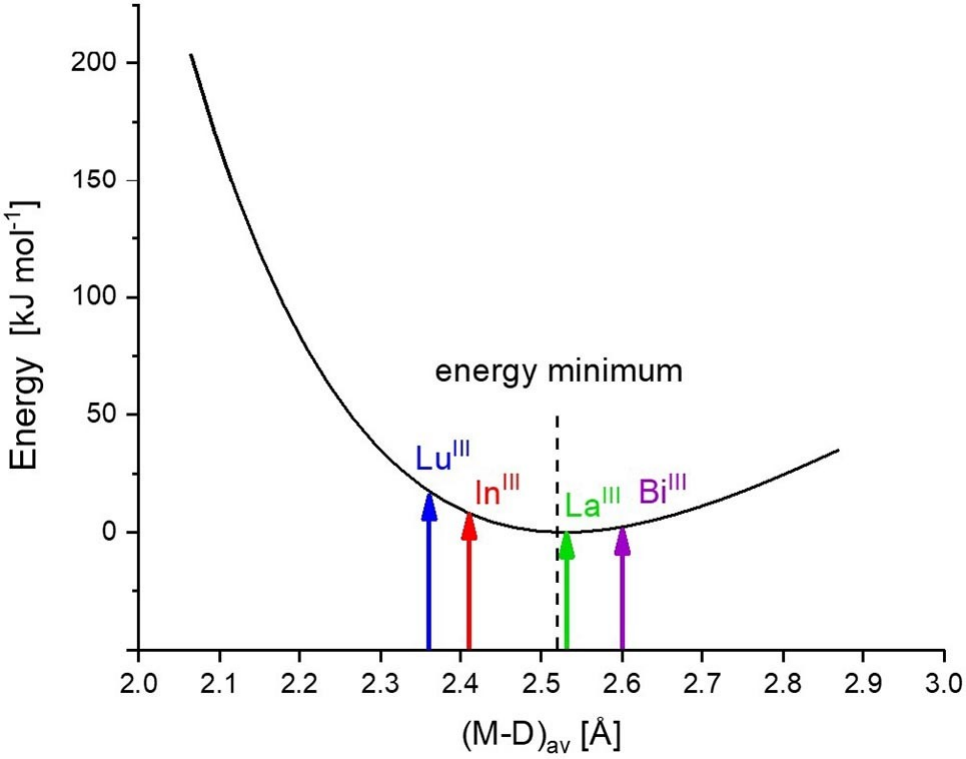
Hole size (and shape) curve for L^4^ (adapted from ref. [21]). The strain energy (MOMEC program and force field^33^, ^34^, ^35^, ^36^) is plotted as a function of the averaged metal–donor distances (M−D)_av_ with the minimum of the curve set to 0 kJ mol^−1^. Apart from the metal‐donor atom−ligand backbone angle deformation, the curve does not include any metal‐ion‐dependent terms. The variation of the metal‐donor atom distances was asymmetric (i. e., the shape of the ligand and its variation were taken into account). The approximation adopted included full geometry optimization of the Zn^II^ complex as a large metal ion and the Co^III^ complex as a small metal ion and linear approximation between these structures to determine the relative changes in metal‐donor atom distance for all eight bonds.

The bispidine cavity is known to be very rigid and, with the basic hexadentate scaffold with four donors attached to C2, C4, N3 and N7, the hexadentate bispidine leads to an elastic coordination sphere,^17^, ^18^, ^28^, ^29^ where larger metal ions prefer relatively high coordination numbers.^18^, ^21^, ^30^ This is also predicted from hole size and shape calculations of hexa‐ and octadentate bispidines using a molecular mechanics‐based approach.^21^, ^31^, ^32^ The ligand‐based strain energy, the “energy penalty” imposed by the ligand, when the metal ion size (average metal‐donor distance) is smaller or larger than the optimum of about 2.5 Å is shown in Figure 2.^31^, ^33^, ^34^, ^35^, ^36^ Eight‐coordinate Bi^III^ has a nearly ideal size (see above) but, as the metal ion is not at the center of the cavity, it might require additional pendant donors to fully encapsulate the metal ion. In a first approach to evaluate the coordination geometry of bismuth(III)‐bispidine complexes, we therefore prepared and characterized the structures of complexes with a series of hepta‐ and octadentate bispidines with mixed amine‐pyridine‐carboxylate donor sets.

It is unsurprising that the heptadentate ligands L^1^, L^2^ and L^3^ lead to unsaturated coordination spheres with Bi^III^ that were completed by anions (bidentate nitrates, L^1^ and L^3^) or crystallize as a dinuclear structure (L^2^) with bridging carboxylates. Interestingly, the structure of the octadentate bispidine L^4^ indicates that the coordination sphere still is unsaturated with a monodentate nitrate coordinated to the complex. In solution, we anticipate that the anions are replaced by solvent molecules, and this is consistent with the solution NMR data (see the Supporting Information). We also assume that the dinuclear structure with L^2^ adopts a mononuclear solution structure similar to those with L^1^ and L^3^, and this also is consistent with the solution spectroscopy (see the Supporting Information). From the structural studies and solution spectroscopy it therefore emerges that nonadentate bispidines might provide an ideal coordination sphere for stable and inert Bi^III^ complexation‐coordination of decadentate bispidines might also be possible and, in terms of stability and inertness then would be preferred.

[Bi^III^(L^4^‘)(NO_3_)]_2_(NO_3_)_2_ (Figure 1 and Table 1) is a dinuclear complex with a partially hydrolyzed ligand, that is, the (aminomethyl)picolinic acid pendant group at N3 is hydrolyzed when the complexation reaction is performed in acidic solution. Structurally, the complex is similar to the others reported here, supporting the observation that the bispidine cavity is suitable for Bi^III^.

Apart from the dinuclear structure with L^2^, all Bi^III^ ions discussed here are nona‐coordinate with N_6_O (L^1^, L^2^), N_5_O_2_ (L^3^) or N_6_O_2_ (L^4^) donor sets of the bispidine and bi‐ or monodentate NO_3_^−^ as co‐ligand (except for the L^2^ based dinuclear structure). The Bi^III^‐O distances in general are approximately 0.1 Å shorter than the Bi^III^‐N distances, and the average Bi^III^‐donor lengths are slightly longer than 2.5 Å, that is, bispidines provide an ideal cavity for Bi^III^ and, based on the structural data, nonadentate bispidines are predicted to be ideal ligands for ^213^Bi^III^ targeted therapy.

### ^213^Bi^III^ labeling

In previous radiolabeling studies, functionalized bispidines were found to be capable of rapidly incorporating various radionuclides into stable and inert chelates. Tetra‐, penta‐ and, in particular, hexadentate ligands proved highly suitable for ^64^Cu^II^,^23^, ^25^, ^37^, ^38^ whereas the octadentate L^4^ and a similar oxine‐based derivative were successfully matched to ^111^In^III^, ^177^Lu^III^ and ^225^Ac^III^.^21^, ^26^

**Figure 3:**
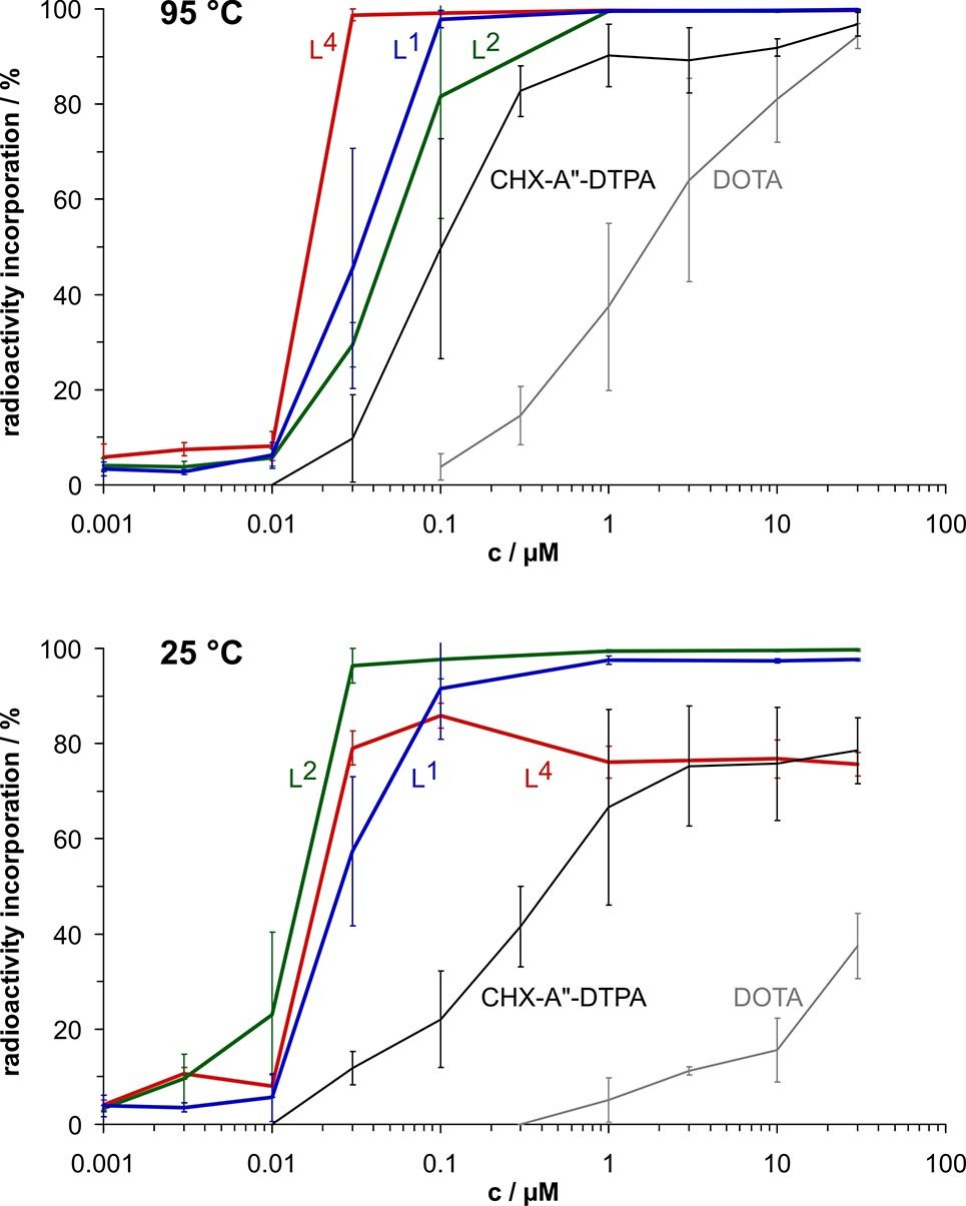
Incorporation of ^213^Bi^III^ by bispidine ligands L^1^, L^2^, and L^4^ as a function of ligand concentration. Radiometal complexation was performed in a total volume of 0.1 mL at pH 5 for 5 min at 95 (top) and 25 °C (bottom); data are given as mean values±SD, *n*=3–6. Literature data for CHX−A′′‐DTPA and DOTA are shown for comparison.^5^

**Figure 4:**
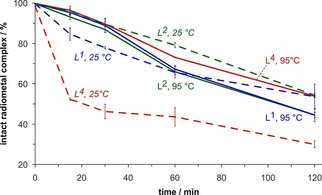
Percentage of intact ^213^Bi^III^ complexes as a function of time upon challenge with 0.1 M aqueous sodium DTPA (pH 7.5, 37 °C). Labeling prior to challenge was done at pH 5 for 5 min and either at 95 °C (solid lines, upright labels) or 25 °C (dashed lines, labels in italics); data are given as mean values±SD, *n*=3.

Accordingly, L^1^, L^2^ and L^4^ show a higher labeling efficiency for ^213^Bi^III^ as compared to the current gold standards CHX−A′′‐DTPA and DOTA (Figure 3). Also, the chelates were found to be quite stable in a transchelation challenge experiment with DTPA as competitor (Figure 4). Whereas the transchelation velocity of the most inert complex with the octadentate ligand, ^213^Bi‐L^4^, is comparable to that of ^213^Bi‐CHX−A′′‐DTPA^5^ (Figure S21), ^213^Bi‐L^1^ and ^213^Bi‐L^2^, that is, the systems with heptadentate bispidines, are somewhat less inert (Figure 4, solid curves). This is because, once the metal ion is coordinated to the open and relatively large cavity of the bispidine platform, it is embraced by the donor side arms and rendered kinetically inert by encapsulation,^15^ yet to a different extent for L^4^ as compared to L^1^ and L^2^ because of the different overall efficiency of encapsulation by the ligands (see structures in Figure 1).

There is an intriguing difference in the observed resistance against transchelation for the chelate ^213^Bi‐L^4^, depending on whether the radiometal complexation was performed at room temperature (RT, ca. 25 °C) or near boiling aqueous solutions (95 °C). The dashed curves in Figure 4 indicate that the labeling product of L^4^ at RT apparently comprises a very labile species, which is readily demetallated within the first minutes, and another complex that seems to be identical to the species obtained by labeling at 95 °C because it is decomposing with the same velocity.

**Figure 5:**
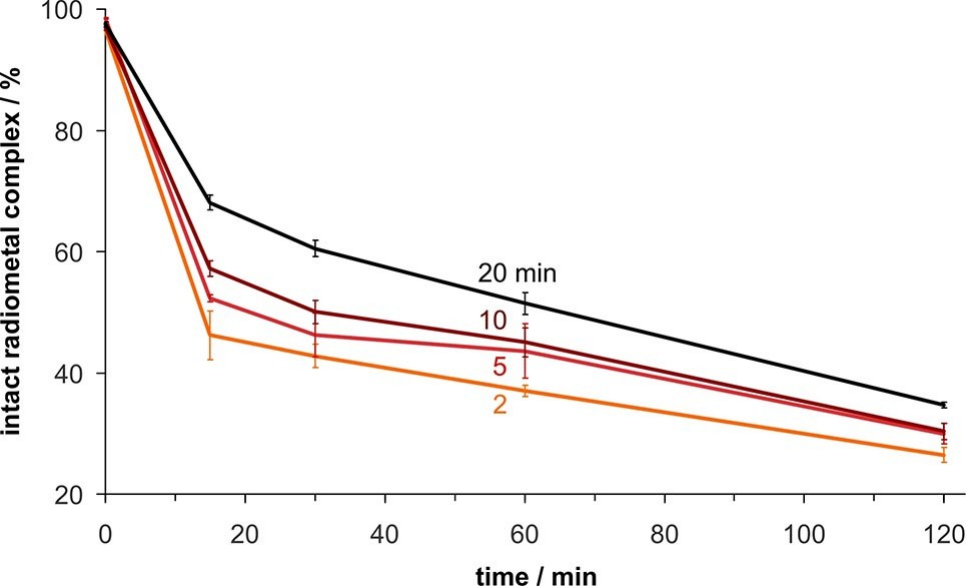
Percentage of intact ^213^Bi^III^‐L^4^ species as functions of time upon challenge with 0.1 M aqueous sodium DTPA (pH 7.5, 37 °C). Labeling prior to challenge was done at pH 5 and 25 °C for different durations (2, 5, 10, and 20 min); data are given as mean values±SD, *n*=3.

Figure 5 shows that the fraction of the labile species decreases with reaction time at RT, indicating that it might be a rapidly forming encounter complex that slowly transforms into the final complex according to Figure 1. Although we can currently not provide any further details about the species involved, we hypothesize that the presence of two distant carboxylates in L^4^ gives rise to a two‐step complexation mechanism, similar to that observed for DOTA‐type ligands.^39^ First, the L^4^ carboxylates replace for example two iodide ligands from the [BiI_5_]^2−^ precursor obtained from the generator, resulting in a [BiI_3_L^4^]^x−^ species with some of the nitrogen donors of the bispidine still protonated. Then, a comparably slow rearrangement at RT and pH 5 delivers the same species obtained instantaneously at 95 °C (Figure 1). No such observations are made for L^1^ and L^2^, because a stable intermediate apparently cannot be formed with only one carboxylate.

### Hydrophilicity

For in‐vivo applications, that is, for the biodistribution and excretion of the radiolabels, a relatively high hydrophilicity of the complexes and bioconjugates generally is of advantage. Usually, this is assessed by the distribution of the radiometal complexes between water and *n*‐octanol at physiological pH (log *D*_7.4_), and the earlier reported ^64^Cu^II^‐bispidine complexes are known to be hydrophilic and to have favorable biodistribution properties.^18^, ^19^, ^21^, ^22^, ^23^, ^24^, ^25^ The experimental data are given in Table 2, where computational data appear for comparison. The computed values are based on charge distributions calculated by a molecular mechanics based approach and a QSAR‐type computation of the log *D*_7.4_ values that has been described previously (modifications of the QSAR fit and parameter values used are given as Supporting Information).^40^, ^41^ Although this procedure is still in a developing stage (primarily also in terms of the scope), it allows to predict the hydrophilicities with an appreciable accuracy. The variation of hydrophilicities of isomeric species (e. g., complexes of ligands L^1^ vs. L^2^, L^5^ vs. L^6^) is qualitatively correctly predicted. Two sets of computed log *D*_7.4_ values are given in Table 2, the second one labeled *all* includes experimental data both from the previous study and from experimental data given here. Of some concern is that, in cases where the coordination number and sphere in solution might differ from that in the solid (i. e., in cases in which the coordination sphere in the solid is completed by a mono‐ or bidentate anion), the prediction is ambiguous. In these cases, modeling of the complex geometry by molecular mechanics or DFT‐based approaches may need to precede the calculation of the log *D*_7.4_ values (see the Supporting Information for computed values with various possible co‐ligands and further discussion).

**Table 2:** Experimental and computed log *D*_7.4_ values of Bi^III^ and Cu^II^ bispidine complexes.^[a]^

Ligand	^64^Cu^II^			^213^Bi^III^		
	exp.	comp.		exp.	comp.	
		pset^[b]^	all^[c]^		pset^[b]^	all^[c]^
L^1^				−1.76±0.05	−2.68	−2.35
L^2^				−2.66±0.08	−2.76	−2.35
L^3^				–	−3.76	−3.43
L^4^				−2.74±0.06	−3.67	−3.09
L^5^	−3.78±0.02^47^	−3.41	−3.36			
L^6^	−2.74±0.02^47^	−3.15	−2.87			
L^7^	−2.77^22^	−2.84	−2.80			
L^8^	−2.84^22^	−2.72	−2.74			
L^9^	−2.44^25^	−2.21	−2.16

[a] See the Supporting Information for a discussion of the problem of labile mono‐ and bidentate co‐ligands and computational details. [b] Parameterization dataset, including Cu^II^‐L^5^+ and Cu^II^‐L^6^ complexes. [c] All experimental data: parameterization dataset and L^1^–L^2^, L^4^–L^9^ complexes.

## Conclusions

The four first‐generation hepta‐ and octadentate bispidines prepared for efficient radiolabeling with ^213^Bi^III^, and their ability to form thermodynamically stable and kinetically inert ^213^Bi^III^ complexes, are the first successful step toward novel agents for targeted radiotherapy with ^213^Bi^III^. As for other bispidines used in radiopharmaceutical applications, these systems are well suited for efficient formation of various bioconjugates.^22^, ^23^, ^24^, ^25^ With three of the four ligands, radiolabeling is significantly faster than with the “gold standard” DOTA, and challenge experiments with an excess of a strong competing ligand (transchelation to DTPA) show that the bispidine complexes are only slightly less inert than those with DOTA. This is not unexpected due to the fact that only hepta‐ and octadentate bispidines were used so far, whereas all these Bi^III^ complexes are shown to be nonacoordinate in the solid state and in solution. The structural work clearly shows that nona‐ or even decadentate bispidines are required to fully encapsulate Bi^III^, and these types of ligands with one or two carboxylate and two or three pyridine groups appended to the tertiary amines to lead to fully encapsulated mono‐ or dicationic Bi^III^ complexes as well as derivatives with linkers to couple the ligands to biological vectors are relatively simple to prepare with bispidine scaffold. Structural modeling as well as the computational methods presented here to compute hydrophilicities will help to optimize these ligands.

## Experimental Section

**Materials and methods**. Chemicals and solvents were purchased from Sigma‐Aldrich, Honeywell, Merck, ABCR, Fisher Scientific, VWR, ACROS or TCI, were of the highest available purity and used without further purification. Deuterated solvents were obtained from Deutero. Milli‐Q water was used for HPLC separations and radiochemical experiments.

**Radio TLC**. iTLC‐SA plates (Agilent) were used as stationary phase and 0.1 M aqueous sodium citrate (pH 5.5) or 0.1 M aqueous DTPA (pH 7.5) as mobile phases. Readout of chromatograms was done using a Bioscan TLC scanner, consisting of B‐MS‐1000 scanner, B‐EC‐1000 detector with a B‐FC‐3600 GM tube.

**Flash column chromatography**. Flash column chromatography was performed on a puriFlash® XS 420 V 1.00 instrument with UV detector (Interchim). Silica gel (pore size 60 Å, particle size 40–63 μm, 230–400 mesh, Honeywell) was used as stationary phase and CH_2_Cl_2_/MeOH or petroleum ether/ethyl acetate as liquid phase.

**NMR spectroscopy**. NMR spectra were recorded at room temperature on Bruker Avance I 200, Bruker Avance II 400 or Bruker Avance III 600 spectrometers. Chemical shifts *δ* of ^1^H and ^13^C (^1^H decoupling) spectra are reported in ppm relative to the solvent signal references (CDCl_3_: *δ*_H_=7.27 ppm, *δ*_C_=77.00 ppm; CD_3_OD: *δ*_H_=3.31 ppm, *δ*_C_=49.15 ppm; CD_3_CN: *δ*_H_=1.94 ppm, *δ*_C_=118.69 ppm; [D_6_]DMSO: *δ*_H_=2.50 ppm, *δ*_C_=39.51 ppm; D_2_O: *δ*_H_=4.75 ppm). Two‐dimensional correlation spectra (COSY, HSQC, HMBC) and ^13^C‐DEPT135 were used for signal assignment.

**Mass spectrometry (MS)**. High‐resolution (HR) mass spectra were recorded on a Bruker ApexQe hybrid 9.4 T FT‐ICR instrument (ESI). Reaction monitoring was conducted on a Waters Acquity+SQD2 UPLC‐MS system. MALDI‐TOF measurements were recorded on a Daltonic Autoflex II TOF/TOF instrument (Bruker) and 3‐hydroxypicolinic acid (HPA) was used as matrix.

**Elemental analysis**. Elemental analyses (CHN) were measured on a vario MIKRO cube instrument (Elementar) by the Microanalysis Laboratory of the Chemical Institutes (Heidelberg University).

**X‐ray crystal structural analysis**. Data were collected on a Bruker AXS Smart 1000 CCD diffractrometer (Mo_Kα_ radiation) or an Agilent Technologies Supernova‐E CCD diffractometer (Mo_Kα_ or Cu_Kα_ radiation). See the Supporting Information for experimental details and crystallographic data tables. Deposition numbers 1916434 (**1**), 1916435 (L^1^), 1916436 (**6**), 1916437 (**7**), 1916438 ([Bi(L^4^)(NO_3_)]H_2_O), 1916439 ([Bi(L^4^′)(NO_3_)]_2_(NO_3_)_2_), 1916440 ([Bi(L^2^)]_2_(NO_3_)_4_⋅4MeOH), 1916441 ([Bi(L^1^)(NO_3_)](NO_3_)MeOH), and 1916442 ([Bi(L^3^)(Br)_0.6_(NO_3_)_0.4_]MeOH) contain the supplementary crystallographic data for this paper. These data are provided free of charge by the joint Cambridge Crystallographic Data Centre and Fachinformationszentrum Karlsruhe Access Structures service www.ccdc.cam.ac.uk/structures.

**Computational work**. The charge distributions and log *D*_7.4_ values as well as the hole size calculations were calculated with the MOMEC program and force field;^33^, ^34^, ^35^, ^36^, ^42^ the methods for the computation of charge distributions and log *D*_7.4_ values have been published before;^40^, ^41^ modifications and parameter sets are given as Supporting Information.

**Syntheses**. Piperidone **P** was prepared according to a literature‐known procedure,^43^ and the synthesis of the octadentate ligand L^4^ was reported before.^21^

*Dimethyl 9‐oxo‐2,4‐di(pyridin‐2‐yl)‐7‐(pyridin‐2‐ylmethyl)‐3,7‐diazabicyclo[3.3.1]nonane‐1,5‐dicarboxylate (**1**)*. **P** (10.0 g, 27.1 mmol, 1.0 equiv) was dissolved in 50 mL EtOH, and the solution was heated to 50 °C. 2‐Picolylamine (3.55 g, 32.8 mmol, 1.2 equiv) and formaldehyde (4.83 mL, 37 % in MeOH/H_2_O, 64.9 mmol, 2.4 equiv) were added dropwise, and the reaction mixture was refluxed for 3 h. The solvent was evaporated, and the remaining solid was recrystallized from *i*PrOH/Et_2_O (1 : 1) yielding **1** as orange crystals (4.90 g, 9.77 mmol, 36 %). ^1^H NMR (600.13 MHz, 22 °C, CDCl_3_): *δ*=3.08 (d, ^2^ *J*_H,H_=11.5 Hz, 2 H, N^7^CH_2,ax/equiv)_, 3.59 (s, 2 H, N^7^CH_2_py), 3.61 (d, ^2^ *J*_H,H_=11.5 Hz, 2 H, N^7^CH_2,ax/equiv)_, 3.73 (s, 6 H, COOCH_3_), 5.16 (s, 2 H, N^3^CH), 7.10 (ddd, ^3^ *J*_H,H_=7.4, 5.0 Hz, ^4^ *J*_H,H_=1.1 Hz, 1 H, pic), 7.12‐7.18 (m, 2 H, py), 7.44 (d, ^3^ *J*_H,H_=7.8 Hz, 1 H, pic), 7.48‐7.56 (m, 3 H, pic, py), 7.64 (td, ^3^ *J*_H,H_=7.7 Hz, ^4^ *J*_H,H_=1.8 Hz, 2 H, py), 8.36 (d, ^3^ *J*_H,H_=4.9 Hz, 1 H, pic), 8.38 (dd, ^3^ *J*_H,H_=4.0 Hz, ^4^ *J*_H,H_=0.7 Hz, 2 H, py) ppm. ^13^C NMR (150.90 MHz, 22 °C CDCl_3_): *δ*=52.1, 57.9, 61.7, 63.1, 66.0, 122.0, 122.6, 123.2, 123.7, 136.1, 136.5, 148.6, 156.9, 157.4, 169.4 ppm. HR‐ESI MS: (pos., MeOH): *m/z* [**1**+Na]^+^ calcd. 524.1904, obsd. 524.1910; [2 **1**+Na]^+^ calcd. 1025.3917, obsd. 1025.3924. Elemental analysis: [**1**⋅H_2_O] calcd. (%): C 62.42, H 5.63, N 13.48; obsd. C 62.45, H 5.59, N 13.6.

*Dimethyl 9‐hydroxy‐2,4‐di(pyridin‐2‐yl)‐7‐(pyridin‐2‐ylmethyl)‐3,7‐diazabicyclo[3.3.1]nonane‐1,5‐dicarboxylate (**2**)*. **1** (3.00 g, 5.98 mmol, 1.0 equiv) was dissolved in a mixture of 1,4‐dioxane and water (3 : 1, 90 mL) and cooled to −5 °C. A solution of NaBH_4_ (0.11 g, 2.99 mmol, 0.5 equiv), in 30 mL 1,4‐dioxane/water (3 : 1) was added dropwise and the reaction mixture was stirred overnight at −2 °C. Concentrated sulphuric acid was added to the mixture to achieve pH 1. After 30 min of stirring at RT, the pH was adjusted to pH 10 with an aqueous solution of sodium hydroxide (20 wt%). The resulting precipitate was removed by filtration and the filtrate was extracted with CH_2_Cl_2_ (3×20 mL). The combined organic phases were dried over Na_2_SO_4_ and evaporated. **2** was obtained as colorless solid with a yield of 59 % (1.78 g, 3.53 mmol). ^1^H NMR (600.13 MHz, 22 °C, CD_3_OD): *δ*=2.73 (d, ^2^ *J*_H,H_=12.1 Hz, 2 H, N^7^CH_2,ax/equiv)_, 2.83 (d, ^2^ *J*_H,H_=11.8 Hz, 2 H, N^7^CH_2,ax/equiv)_, 3.54 (s, 2 H, N^7^CH_2_py), 3.65 (s, 6 H, COOCH_3_), 4.58 (d, ^4^ *J*_H,H_=1.3 Hz, 2 H, N^3^CH), 4.80 (s, 1 H, CHOH), 7.19‐7.26 (m, 4 H, py), 7.38‐7.42 (m, 1 H, pic), 7.68 (td, ^3^ *J*_H,H_=7.7 Hz, ^4^ *J*_H,H_=1.8 Hz, 2 H, py), 7.96 (td, ^3^ *J*_H,H_=7.6 Hz, ^4^ *J*_H,H_=1.8 Hz, 1 H, pic), 8.00‐8.02 (m, 1 H, pic), 8.43–8.46 (m, 3 H, py, pic) ppm. ^13^C NMR (150.90 MHz, 22 °C, CD_3_OD): *δ*=50.5, 52.5, 53.4, 65.9, 67.4, 74.9, 123.5, 124.1, 124.2, 127.1, 138.0, 138.7, 149.2, 150.1, 158.6, 159.4, 174.4 ppm. HR‐ESI MS (pos., MeOH): *m/z* [**2**+H]^+^ calcd. 504.2241, obsd. 504.2243. Elemental analysis: [**2**] calcd. (%) :C 64.40, H 5.81, N 13.91; obsd. C 64.44, H 5.80, N 14.05.

Dimethyl 3‐((6‐(tert‐butoxycarbonyl)pyridin‐2‐yl)methyl)‐9‐hydroxy‐2,4‐di(pyridin‐2‐yl)‐7‐(pyridin‐2‐ylmethyl)‐3,7‐diazabicyclo[3.3.1]nonane‐1,5‐dicarboxylate (**3**). **2** (0.40 g, 0.79 mmol, 1.0 equiv) was dissolved in 50 mL MeCN. tert‐Butyl‐6‐(bromomethyl)picolinate (0.22 g, 0.79 mmol, 1.0 equiv) and Na_2_CO_3_ (0.51 g, 4.77 mmol, 6.0 equiv) were added and the reaction mixture was refluxed for 24 h. Excess Na_2_CO_3_ was removed by filtration and the filtrate was evaporated. The remaining solid was dissolved in 50 mL CH_2_Cl_2_ and 50 mL H_2_O. The aqueous phase was extracted with CH_2_Cl_2_ and the combined organic phases were dried over Na_2_SO_4_. The solvent was evaporated and the crude product recrystallized from ethyl acetate with a yield of 83 % (0.46 g, 0.66 mmol). ^1^H NMR (600.13 MHz, 22 °C, CD_3_OD): δ=1.64 (s, 9 H, COOtBu), 2.53 (d, ^2^J_H,H_=12.2 Hz, 2 H, N^7^CH_2,ax/equiv)_, 2.66 (d, ^2^J_H,H_=12.2 Hz, 2 H, N^7^CH_2,ax/equiv)_, 3.56 (s, 2 H, N^7^CH_2_py), 3.58 (s, 2 H, N^3^CH_2_pa), 3.60 (s, 6 H, COOCH_3_), 4.74 (s, 2 H, N^3^CH), 4.88 (s, 1 H, CHOH), 7.03 (br d, ^3^J_H,H_=6.8 Hz, 1 H, pa), 7.06–7.12 (m, 2 H, py), 7.42 (td, ^3^J_H,H_=7.6 Hz, ^4^J_H,H_=1.3 Hz, 2 H, py), 7.46 (dd, ^3^J_H,H_=6.8, 5.3 Hz, 1 H, pic), 7.56 (t, ^3^J_H,H_=7.7 Hz, 1 H, pa), 7.64 (d, ^3^J_H,H_=7.7 Hz, 1 H, pa), 7.74 (br d, ^3^J_H,H_=4.7 Hz, 1 H, pic), 7.91 (d, ^3^J_H,H_=7.6 Hz, 2 H, py), 7.97 (td, ^3^J_H,H_=7.7 Hz, ^4^J_H,H_=1.5 Hz, 1 H, pic), 8.24 (d, ^3^J_H,H_=4.0 Hz, 2 H, py), 8.60 (d, ^3^J_H,H_=4.1 Hz, 1 H, pic) ppm. ^13^C NMR (150.90 MHz, 22 °C, CD_3_OD): δ=28.6, 50.7, 52.6, 54.8, 61.9, 66.2, 72.8, 74.9, 83.1, 123.8, 124.2, 124.4, 126.8, 127.4, 127.8, 137.6, 138.0, 138.8, 149.0, 150.0, 159.0, 160.1, 160.3, 165.6, 173.6 ppm. HR‐ESI MS (pos., MeOH): m/z [**3**+H]^+^ calcd. 695.3188, obsd. 695.3191. Elemental analysis: [**3**⋅0.5 H_2_O] calcd. (%): C 64.85, H 6.16, N 11.94; obsd. C 64.78, H 6.11, N 11.81.

6‐((9‐Hydroxy‐1,5‐bis(methoxycarbonyl)‐2,4‐di(pyridin‐2‐yl)‐7‐(pyridin‐2‐ylmethyl)‐3,7‐diazabicyclo[3.3.1]nonan‐3‐yl)methyl)picolinic acid (L^2^⋅H(TFA)). **3** (0.90 g, 1.30 mmol, 1.0 equiv) was dissolved in 7 mL CH_2_Cl_2_ and 7 mL H(TFA) and stirred overnight at RT. The solvent was evaporated and L^2^ recrystallized from MeCN as TFA salt with a yield of 63 % (0.61 g, 0.81 mmol). ^1^H NMR (600.13 MHz, 22 °C, CD_3_OD): δ=3.55 (s, 2 H, N^3^CH_2_pa), 3.60 (s, 6 H, COOCH_3_), 3.71 (d, ^2^J_H,H_=12.3 Hz, 2 H, N^7^CH_2,ax/equiv)_, 4.18 (d, ^2^J_H,H_=12.2 Hz, 2 H, N^7^CH_2,ax/equiv)_, 4.67 (s, 2 H, N^7^CH_2_py), 4.88 (s, 1 H, CHOH), 4.98 (s, 2 H, N^3^CH), 7.18–7.25 (m, 3 H, pa, py), 7.34 (br d, ^3^J_H,H_=6.0 Hz, 2 H, py), 7.66 (dd, ^3^J_H,H_=7.2, 4.9 Hz, 1 H, pic), 7.72 (t, ^3^J_H,H_=7.6 Hz, 2 H, py), 7.78‐7.85 (m, 2 H, pic, pa), 7.93 (d, ^3^J_H,H_=7.5 Hz, 1 H, pa), 8.06 (t, ^3^J_H,H_=7.5 Hz, 1 H, pa), 8.18 (br d, ^3^J_H,H_=3.2 Hz, 2 H, py), 8.87 (br d, ^3^J_H,H_=4.2 Hz, 1 H, pic) ppm. ^13^C NMR (150.90 MHz, 22 °C, CD_3_OD): δ=51.0, 53.4, 54.3, 57.2, 63.5, 72.3, 72.4, 124.5, 125.4, 126.2, 127.2, 127.3, 128.4, 138.9, 139.4, 140.2, 148.3, 151.0, 151.2, 151.7, 156.3, 158.3, 167.0, 170.5 ppm. HR‐ESI MS (pos., MeOH): m/z [L^2^+H]^+^ calcd. 639.2562, obsd. 639.2578. Elemental analysis: [L^2^⋅H(TFA)⋅0.5 MeCN] calcd. (%): C 57.47, H 4.76, N 11.77; obsd. C 57.20, H 4.78, N 11.66.

Dimethyl 7‐((6‐(tert‐butoxycarbonyl)pyridin‐2‐yl)methyl)‐9‐hydroxy‐2,4‐di(pyridin‐2‐yl)‐3‐(pyridin‐2‐ylmethyl)‐3,7‐diazabicyclo[3.3.1]nonane‐1,5‐dicarboxylate (**5**). The unprotected secondary amine precursor **4** was obtained as described in the literature.^44^, ^45^ **4** (1.74 g, 2.38 mmol, 1.0 equiv) was dissolved in 100 mL MeCN. tert‐Butyl‐6‐(bromomethyl)picolinate (0.65 g, 2.38 mmol, 1.0 equiv) and Na_2_CO_3_ (2.52 g, 23.8 mmol, 10 equiv) were added, and the reaction mixture was refluxed for 24 h. Excess Na_2_CO_3_ was removed by filtration and the filtrate was evaporated. The remaining solid was dissolved in 50 mL CH_2_Cl_2_ and 50 mL H_2_O. The aqueous phase was extracted with CH_2_Cl_2_ and the combined organic phases were dried over Na_2_SO_4_. The solvent was evaporated and the crude product recrystallized from MeCN with a yield of 95 % (1.57 g, 2.26 mmol, 0.95 equiv). ^1^H NMR (600.13 MHz, 22 °C, CD_3_CN): δ = 1.60 (s, 9 H, COOtBu), 2.30‐2.40 (m, 4 H, N^7^CH_2,ax/equiv)_, 3.44 (s, 2 H, N^7^CH_2_pa), 3.49 (s, 2 H, N^3^CH_2_py), 3.55 (s, 6 H, COOCH_3_), 4.38 (br s, 1 H, CHOH), 4.82 (s, 2 H, N^3^CH), 6.61 (d, ^3^J_H,H_=7.7 Hz, 1 H, pic), 7.11–7.17 (m, 3 H, py, pic), 7.48 (td, ^3^J_H,H_=7.6 Hz, ^4^J_H,H_=1.6 Hz, 2 H, py), 7.52 (td, ^3^J_H,H_=7.7 Hz, ^4^J_H,H_=1.7 Hz, 1 H, pic), 7.61 (br d, ^3^J_H,H_=6.3 Hz, 1 H, pa), 7.92 (t, ^3^J_H,H_=7.7 Hz, 1 H, pa), 8.02 (d, ^3^J_H,H_=7.8 Hz, 1 H, pa), 8.06 (br d, ^3^J_H,H_=7.7 Hz, 2 H, py), 8.37 (br d, ^3^J_H,H_=3.9 Hz, 2 H, py), 8.48 (br d, ^3^J_H,H_=4.0 Hz, 1 H, pic) ppm. ^13^C NMR (150.90 MHz, 22 °C, CD_3_CN): δ=28.5, 50.4, 53.8, 57.1, 65.9, 70.6, 72.5, 82.6, 123.1, 123.4, 124.4, 125.1, 125.7, 128.6, 136.7, 136.8, 138.4, 149.2, 150.0, 150.6, 157.9, 159.2, 160.9, 165.7, 173.3 ppm. HR‐ESI MS (pos., MeOH): m/z [**5**+H]^+^ calcd. 695.3188, obsd. 695.3199; [**5**+Na]^+^ calcd. 717.3007, obsd. 717.3019. Elemental analysis: [**5**⋅0.5 H_2_O] calcd. (%): C 64.85, H 6.16, N 11.94; obsd. C 64.62, H 6.30, N 11.89.

6‐((9‐Hydroxy‐1,5‐bis(methoxycarbonyl)‐6,8‐di(pyridin‐2‐yl)‐7‐(pyridin‐2‐ylmethyl)‐3,7‐diazabicyclo[3.3.1]nonan‐3‐yl)methyl)picolinic acid (L^1^). **5** (1.57 g, 2.26 mmol, 1.0 equiv) was dissolved in 9 mL CH_2_Cl_2_ and 9 mL H(TFA) and stirred overnight at RT. The solvent was evaporated and L^1^ recrystallized from iPrOH as double TFA salt with a yield of 66 % (1.30 g, 1.50 mmol). ^1^H NMR (600.13 MHz, 22 °C, CD_3_OD): δ = 3.64 (s, 6 H, COOCH_3_), 3.70 (s, 2 H, N^3^CH_2_py), 3.78 (d, ^2^J_H,H_=12.7 Hz, 2 H, N^7^CH_2,ax/equiv)_, 3.98 (d, ^2^J_H,H_=12.4 Hz, 2 H, N^7^CH_2,ax/equiv)_, 4.78 (s, 2 H, N^7^CH_2_pa), 4.95 (s, 1 H, CHOH), 4.99 (s, 2 H, N^3^CH), 7.24 (dd, ^3^J_H,H_=7.5, 4.9 Hz, 2 H, py), 7.27‐7.36 (m, 3 H, py, pic), 7.53‐7.60 (m, 1 H, pic), 7.76 (td, ^3^J_H,H_=7.6 Hz, ^4^J_H,H_=1.3 Hz, 2 H, py), 7.99 (d, ^3^J_H,H_=7.7 Hz, 1 H, pa), 8.04–8.12 (m, 3 H, py, pic), 8.25 (t, ^3^J_H,H_=7.7 Hz, 1 H, pa), 8.43 (d, ^3^J_H,H_=7.9 Hz, 1 H, pa), 8.69 (br d, ^3^J_H,H_=5.0 Hz, 1 H, pic) ppm. ^13^C NMR (150.90 MHz, 22 °C, CD_3_OD): δ=50.9, 53.6, 54.5, 57.0, 63.1, 71.9, 72.7, 125.4, 125.9, 127.1, 127.5, 131.0, 139.5, 141.0, 144.7, 151.0, 151.1, 151.5, 155.9, 156.4, 167.7, 170.3 ppm. HR‐ESI MS (pos., MeOH): m/z [L^1^+H]^+^ calcd. 639.2562, obsd. 639.2563; [L^1^+K]^+^ calcd. 677.2121, obsd. 677.2031. Elemental analysis: [L^1^⋅2 H(TFA)⋅2 iPrOH⋅0.5 H_2_O] calcd. (%): C 53.06, H 5.36, N 8.44; obsd. C 53.00, H 5.30, N 8.43.

The N7‐dimethoxybenzene‐substituted precursors **6** and **7** were obtained as described in the literature.^21^

Dimethyl 3‐((6‐(tert‐butoxycarbonyl)pyridin‐2‐yl)methyl)‐7‐(2,4‐dimethoxybenzyl)‐9‐hydroxy‐2,4‐di(pyridin‐2‐yl)‐3,7‐diazabicyclo[3.3.1]nonane‐1,5‐dicarboxylate (**8**). **8** was synthesized according to a slightly modified literature procedure.^46^ **7** (4.00 g, 7.11 mmol, 1.0 equiv) was dissolved in 250 mL MeCN. tert‐Butyl‐6‐(bromomethyl)picolinate (2.32 g, 8.53 mmol, 1.2 equiv) and Na_2_CO_3_ (4.52 g, 42.7 mmol, 6.0 equiv) were added and the reaction mixture was refluxed for 16 h. Excess Na_2_CO_3_ was removed by filtration and **8** was recrystallized from acetone with a yield of 88 % (4.69 g, 6.22 mmol). ^1^H NMR (600.13 MHz, 22 °C, CD_3_CN): δ=1.71 (s, 9 H, COOtBu), 2.37 (d, ^2^J_H,H_=12.0 Hz, 2 H, N^7^CH_2,ax/equiv)_, 2.81 (d, ^2^J_H,H_=12.2 Hz, 2 H, N^7^CH_2,ax/equiv)_, 3.46 (s, 2 H, N^7^CH_2_Ar), 3.63 (s, 6 H, COOCH_3_), 3.66 (s, 2 H, N^3^CH_2_pa), 3.83 (s, 3 H, OCH_3_), 4.33 (s, 3 H, OCH_3_), 4.52 (s, 2 H, N^3^CH), 4.67 (br d, ^3^J_H,H_=6.0 Hz, 1 H, CHOH), 6.53 (dd, ^3^J_H,H_=8.4 Hz, ^4^J_H,H_=2.2 Hz, 1 H, Ar), 6.78 (br d, ^3^J_H,H_=8.0 Hz, 1 H, pa), 6.97 (br d, ^4^J_H,H_=2.1 Hz, 1 H, Ar), 6.99–7.05 (m, 4 H, py), 7.07 (br d, ^3^J_H,H_=8.3 Hz, 1 H, Ar), 7.37 (br t, ^3^J_H,H_=7.7 Hz, 1 H, pa), 7.45 (br d, ^3^J_H,H_=7.5 Hz, 1 H, pa), 7.53 (td, ^3^J_H,H_=7.6 Hz, ^4^J_H,H_=1.5 Hz, 2 H, py), 8.01 (br d, ^3^J_H,H_=3.9 Hz, 2 H, py) ppm. ^13^C NMR (150.90 MHz, 22 °C, CD_3_CN): δ=28.4, 50.1, 53.0, 55.2, 56.3, 59.7, 60.1, 65.4, 72.7, 74.3, 84.1, 102.5, 107.3, 118.9, 123.3, 124.5, 125.9, 126.6, 134.4, 138.5, 138.9, 147.8, 150.1, 158.0, 159.0, 159.9, 162.1, 165.7, 172.5 ppm. HR‐ESI MS (pos., MeOH): m/z [**8**+H]^+^ calcd. 754.3447, obsd. 754.3455. Elemental analysis: [**8**⋅Na_2_CO_3_⋅1.5 H_2_O] calcd. (%): C 56.88, H 5.68, N 7.90; obsd. C 57.02, H 5.68, N 7.96.

*6‐((9‐Hydroxy‐1,5‐bis(methoxycarbonyl)‐2,4‐di(pyridin‐2‐yl)‐3,7‐diazabicyclo[3.3.1]nonan‐3‐yl)methyl)picolinic acid (**9**)*. **9** was synthesized according to a slightly modified literature procedure.^46^ **8** (1.40 g, 1.86 mmol, 1.0 equiv) was dissolved in 13 mL CH_2_Cl_2_. H(TFA) (13 mL) was slowly added and the reaction mixture was stirred overnight at 50 °C. The solvent was evaporated and **9** recrystallized from acetone as double TFA salt (0.97 g, 1.25 mmol, 67 %). ^1^H NMR (600.13 MHz, 22 °C, CD_3_OD): *δ*=3.54 (d, ^2^ *J*_H,H_=13.0 Hz, 2 H, N^7^CH_2,ax/equiv)_, 3.65 (s, 6 H, COOCH_3_), 3.73 (s, 2 H, N^3^CH_2_pa), 3.85 (d, ^2^ *J*_H,H_=12.4 Hz, 2 H, N^7^CH_2,ax/equiv)_, 5.09 (s, 2 H, N^3^CH), 6.87 (br d, ^3^ *J*_H,H_=5.8 Hz 1 H, pa), 7.35 (dd, ^3^ *J*_H,H_=6.9, 5.1 Hz, 2 H, py), 7.43 (br s, 2 H, py), 7.85 (t, ^3^ *J*_H,H_=7.6 Hz, 2 H, py), 7.90 (br t, ^3^ *J*_H,H_=6.9 Hz, 1 H, pa), 8.09 (br d, ^3^ *J*_H,H_=7.2 Hz, 1 H, pa), 8.50 (br d, ^3^ *J*_H,H_=4.0 Hz, 2 H, py) ppm. ^13^C NMR (150.90 MHz, 22 °C, CD_3_OD): *δ*=42.1, 53.3, 53.4, 56.6, 72.4, 125.2, 125.9, 127.3, 128.0, 139.7, 143.9, 151.0, 156.4, 157.4, 170.9 ppm. HR‐ESI MS (pos., MeOH): *m/z* [**9**+H]^+^ calcd. 548.2140, obsd. 548.2153; [**9**+Na]^+^ calcd. 570.1959, obsd. 570.1974; [**9**+K]^+^ calcd. 586.1699, obsd. 586.1625. Elemental analysis: [**9**⋅2 H(TFA)⋅0.5 H_2_O] calcd. (%): C 48.99, H 4.11, N 8.93; obsd. C 49.12, H 4.42, N 8.89.

6‐((7‐(Carboxymethyl)‐9‐hydroxy‐1,5‐bis(methoxycarbonyl)‐2,4‐di(pyridin‐2‐yl)‐3,7‐diazabicyclo[3.3.1]nonan‐3‐yl)methyl)picolinic acid (L^3^). **9**⋅2 H(TFA) (0.96 g, 1.24 mmol, 1.0 equiv) was dissolved in 100 mL MeOH. 3‐Bromoacetic acid (0.17 g, 1.24 mmol, 1.0 equiv) and Na_2_CO_3_ (0.79 g, 7.43 mmol, 6.0 equiv) were added, and the reaction mixture was refluxed overnight. Excess Na_2_CO_3_ was removed by filtration and the product was recrystallized from MeOH with impurities of Na_2_CO_3_ and NaBr a yield of 96 % (0.97 g, 1.19 mmol). ^1^H NMR (600.13 MHz, 22 °C, CD_3_OD): δ=2.49 (d, ^2^J_H,H_=11.8 Hz, 2 H, N^7^CH_2,ax/equiv)_, 2.98 (s, 2 H, N^7^CH_2_COOH), 3.06 (d, ^2^J_H,H_=11.5 Hz, 2 H, N^7^CH_2,ax/equiv)_, 3.58 (s, 6 H, COOCH_3_), 3.60 (s, 2 H, N^3^CH_2_pa), 4.47 (s, 2 H, N^3^CH), 4.78 (s, 1 H, CHOH), 6.47 (d, ^3^J_H,H_=6.9 Hz, 1 H, pa), 7.03 (br s, 2 H, py), 7.11 (br s, 2 H, py), 7.22 (t, ^3^J_H,H_=7.7 Hz, 1 H, pa), 7.44 (d, ^3^J_H,H_=7.4 Hz, 1 H, pa), 7.55 (br s, 2 H, py), 8.41 (br s, 2 H, py) ppm. ^13^C NMR (150.90 MHz, 22 °C, CD_3_OD): δ=50.6, 52.7, 56.1, 65.8, 67.3, 73.5, 122.0, 123.7, 124.5, 125.9, 138.1, 151.1, 154.4, 158.4, 173.5, 178.8 ppm. HR‐ESI MS (neg., MeOH): m/z [L^3^‐H]^−^ calcd. 604.2049, obsd. 604.2054. Elemental analysis: [L^3^⋅Na_2_CO_3_⋅NaBr⋅H_2_O] calcd. (%): C 44.73, H 4.00, N 8.41; obsd. C 44.56, H 3.95, N 8.40.

*[Bi^III^(L^2^)](NO_3_)_2_*. L^2^⋅H(TFA) (100 mg, 0.13 mmol, 1.0 equiv) and Bi(NO_3_)_3_⋅5 H_2_O (64 mg, 0.13 mmol, 1.0 equiv) were dissolved in 30 mL MeOH (pH 4) and stirred at overnight at RT. The complex was recrystallized from MeOH (120 mg, 0.12 mmol, 93 %). ^1^H NMR (399.89 MHz, 22 °C, D_2_O): *δ*=3.27 (d, ^2^ *J*_H,H_=13.7 Hz, 2 H, N^7^CH_2,ax/equiv)_, 3.54 (d, ^2^ *J*_H,H_=13.7 Hz, 2 H, N^7^CH_2,ax/equiv)_, 3.73 (s, 6 H, COOCH_3_), 4.52 (s, 2 H, NCH_2_Ar), 4.85 (s, 2 H, NCH_2_Ar), 5.23 (s, 1 H, CHOH), 5.85 (s, 2 H, N^3^CH), 7.37 (d, ^3^ *J*_H,H_=7.7 Hz, 1 H, pa), 7.47 (br t, ^3^ *J*_H,H_=6.4 Hz, 2 H, py), 7.60 (d, ^3^ *J*_H,H_=7.8 Hz, 2 H, py), 7.70 (d, ^3^ *J*_H,H_=7.7 Hz, 1 H, pic), 7.77–7.83 (m, 2 H, pa, pic), 7.92‐8.02 (m, 3 H, pa, py), 8.09 (td, ^3^ *J*_H,H_=7.7 Hz, ^4^ *J*_H,H_=1.5 Hz, 1 H, pic), 8.85 (d, ^3^ *J*_H,H_=5.0 Hz, 2 H, py), 9.45 (d, ^3^ *J*_H,H_=4.6 Hz, 1 H, pic) ppm. ^13^C NMR (100.55 MHz, 22 °C, D_2_O): *δ*=49.2, 53.7, 53.8, 64.3, 64.9, 70.3, 74.7, 125.6, 126.0, 126.3, 126.4, 127.0, 141.3, 142.1, 142.3, 147.7, 149.0, 152.3, 154.5, 155.4, 170.2, 171.8 ppm. HR‐ESI MS (pos., MeOH): *m/z* [[Bi(L^2^)](NO_3_)]^+^ calcd. 908.2087, obsd. 908.2117; [[Bi(B7)] (OMe)]^+^ calcd. 877.2393, obsd. 877.2427. Elemental analysis: [[Bi(L^2^)](NO_3_)_2_⋅3 H_2_O] calcd. (%): C 39.85, H 3.84, N 10.94; obsd. C 39.70, H 3.90, N 11.24.

*[Bi^III^(L^1^)(NO_3_)](NO_3_)*. L^1^⋅2 H(TFA) (200 mg, 0.20 mmol, 1.0 equiv) and Bi(NO_3_)_3_⋅5 H_2_O (98 mg, 0.20 mmol, 1.0 equiv) were dissolved in 30 mL MeOH (pH 4) and stirred overnight at RT. The complex was recrystallized from MeOH (195 mg, 0.20 mmol, 99 %). ^1^H NMR (600.13 MHz, 22 °C, D_2_O): *δ*=3.11 (d, ^2^ *J*_H,H_=13.2 Hz, 1 H, N^7^CH_2,ax/equiv)_, 3.43‐3.55 (m, 3 H, N^7^CH_2,ax/equiv)_, 3.61 (s, 3 H, COOCH_3_), 3.82 (s, 3 H, COOCH_3_), 4.40 (d, ^2^ *J*_H,H_=15.9 Hz, 1 H, N^3^CH_2_py), 4.52 (d, ^2^ *J*_H,H_=14.8 Hz, 1 H, N^7^CH_2_pa), 4.64 (d, ^2^ *J*_H,H_=14.8 Hz, 1 H, N^7^CH_2_pa), 4.71 (d, ^2^ *J*_H,H_=17.8 Hz, 1 H, N^3^CH_2_py), 5.20 (s, 1 H, CHOH), 5.68 (br s, 2 H, N^3^CH), 7.07 (d, ^3^ *J*_H,H_=7.8 Hz, 1 H, pic), 7.17 (br s, 1 H, py), 7.25–7.28 (m, 1 H, pic), 7.43 (br d, ^3^ *J*_H,H_=6.5 Hz, 1 H, py), 7.61 (td, ^3^ *J*_H,H_=7.7 Hz, ^4^ *J*_H,H_=1.3 Hz, 2 H, pic, py), 7.64 (br d, ^3^ *J*_H,H_=6.4 Hz, 1 H, py), 7.77 (br s, 1 H, py), 7.96 (d, ^3^ *J*_H,H_=7.7 Hz, 1 H, pa), 8.09 (br s, 1 H, py), 8.14 (br s, 1 H, py), 8.27 (d, ^3^ *J*_H,H_=7.8 Hz, 1 H, pa), 8.36 (t, ^3^ *J*_H,H_=7.8 Hz, 1 H, pa), 9.02 (d, ^3^ *J*_H,H_=5.3 Hz, 1 H, pic), 9.07 (br s, 1 H, py) ppm. ^13^C NMR (150.90 MHz, 22 °C, D_2_O): *δ*=49.5, 49.8, 53.5, 54.1, 64.3, 65.6, 70.5, 74.3, 74.7, 124.2, 124.5, 125.4, 126.1, 126.6, 126.8, 128.4, 140.4, 141.1, 141.7, 142.7, 147.9, 148.2, 150.8, 154.7, 155.8, 170.8, 172.7 ppm. HR‐ESI MS (pos., MeOH): *m/z* [[Bi(L^1^)](OMe)]^+^ calcd. 877.2393, obsd. 877.2423, [[Bi(L^1^)(NO_3_)]]^+^ calcd. 908.2087, obsd. 908.2114. Elemental analysis: [[Bi(L^1^)(NO_3_)](NO_3_)⋅2 H_2_O] calcd. (%): C 40.57, H 3.70, N 11.13; obsd. C 40.37, H 3.65, N 10.94.

*[Bi^III^(L^3^)(Br)*_*0.5*_ *(NO_3_)*_*0.5*_ *]* (note that the stoichiometry is slightly different from that of the crystals analyzed by X‐ray diffraction). L^3^ (100 mg, 0.17 mmol, 1 equiv) and Bi(NO_3_)_3_⋅5 H_2_O (80 mg, 0.17 mmol, 1 equiv) were dissolved in MeOH (pH 4) and stirred overnight at RT. The complex was recrystallized from MeOH (87 mg, 0.10 mmol, 60 %). ^1^H NMR (600.13 MHz, 22 °C, [D_6_]DMSO): *δ*=2.86 (d, ^2^ *J*_H,H_=12.8 Hz, 2 H, N^7^CH_2,ax/equiv)_, 3.20 (d, ^2^ *J*_H,H_=12.9 Hz, 2 H, N^7^CH_2,ax/equiv)_, 3.63 (s, 6 H, COOCH_3_), 3.71 (s, 2 H, N^7^CH_2_COO^−^), 4.52 (s, 2 H, N^3^CH_2_pa), 4.92 (d, ^3^ *J*_H,H_=6.1 Hz, 1 H, CHOH), 5.44 (s, 2 H, N^3^CH), 6.78 (d, ^3^ *J*_H,H_=6.1 Hz, 1 H, OH), 7.22 (d, ^3^ *J*_H,H_=7.9 Hz, 1 H, pa), 7.39 (d, ^3^ *J*_H,H_=7.8 Hz, 2 H, py), 7.51–7.55 (m, 2 H, py), 7.57 (d, ^3^ *J*_H,H_=7.6 Hz, 1 H, pa), 7.85 (t, ^3^ *J*_H,H_=7.7 Hz, 1 H, pa), 7.99 (td, ^3^ *J*_H,H_=7.7 Hz, ^4^ *J*_H,H_=1.6 Hz, 2 H, py), 8.88 (d, ^3^ *J*_H,H_=4.3 Hz, 2 H, py) ppm. ^13^C NMR (150.90 MHz, 22 °C, [D_6_]DMSO): *δ*=48.6, 52.9, 53.7, 63.5, 64.3, 70.0, 73.5, 124.1, 124.8, 125.2, 125.6, 140.8, 149.1, 149.8, 153.1, 154.4, 168.0, 169.5, 172.9 ppm. HR‐ESI MS (pos., MeOH): *m/z* [[Bi(L^3^)]]^+^ calcd. 812.1764, obsd. 812.1783. Elemental analysis: [[Bi(L^3^)(Br)_0.5_(NO_3_)_0.5_]⋅MeOH⋅2 H_2_O] calcd. (%): C 39.13, H 3.92, N 8.10; obsd. C 38.98, H 4.02, N 8.39.

*[Bi^III^(L^4^)(NO_3_)]*. L^4^⋅H(TFA) (100 mg, 0.13 mmol, 1.0 equiv) and Bi(NO_3_)_3_⋅5 H_2_O (61 mg, 0.13 mmol, 1.0 equiv) were dissolved in 15 mL of MeOH/H_2_O (1 : 2). The reaction mixture (pH 3) was adjusted to pH 5 with 0.1 M aqueous NaOH solution and heated to 60 °C for 2 h. The solvent was evaporated and the residue recrystallized from hot MeOH (113 mg, 0.12 mmol, 95 %). ^1^H NMR (600.13 MHz, 22 °C, [D_6_]DMSO): *δ*=2.46 (d, ^2^ *J*_H,H_=12.7 Hz, 1 H, N^7^CH_2,ax/equiv)_, 2.90 (d, ^2^ *J*_H,H_=12.2 Hz, 1 H, N^7^CH_2,ax/equiv)_, 3.09 (d, ^2^ *J*_H,H_=12.6 Hz, 1 H, N^7^CH_2,ax/equiv)_, 3.17 (d, ^2^ *J*_H,H_=12.8 Hz, 1 H, N^7^CH_2,ax/equiv)_, 3.64 (s, 3 H, COOCH_3_), 3.66 (s, 3 H, COOCH_3_), 4.00 (d, ^2^ *J*_H,H_=14.6 Hz, 1 H, NCH_2_pa), 4.20 (d, ^2^ *J*_H,H_=17.9 Hz, 1 H, NCH_2_pa), 4.49 (d, ^2^ *J*_H,H_=18.3 Hz, 1 H, NCH_2_pa), 4.60 (d, ^2^ *J*_H,H_=14.7 Hz, 1 H, NCH_2_pa), 4.95 (d, ^3^ *J*_H,H_=5.7 Hz, 1 H, CHOH), 5.23 (s, 1 H, N^3^CH), 5.28 (s, 1 H, N^3^CH), 6.73 (d, ^3^ *J*_H,H_=5.5 Hz, 1 H, OH), 7.08 (d, ^3^ *J*_H,H_=8.0 Hz, 1 H, pa), 7.31 (d, ^3^ *J*_H,H_=7.4 Hz, 1 H, py), 7.34 (d, ^3^ *J*_H,H_=7.8 Hz, 1 H, py), 7.38 (t, ^3^ *J*_H,H_=6.4 Hz, 1 H, py), 7.44 (t, ^3^ *J*_H,H_=6.1 Hz, 1 H, py), 7.68 (d, ^3^ *J*_H,H_=7.5 Hz, 1 H, pa), 7.83–7.97 (m, 4 H, pa, py, pa, py), 8.05 (d, ^3^ *J*_H,H_=7.3 Hz, 1 H, pa), 8.29 (t, ^3^ *J*_H,H_=7.6 Hz, 1 H, pa), 8.49 (d, ^3^ *J*_H,H_=4.8 Hz, 1 H, py), 8.79 (d, ^3^ *J*_H,H_=5.0 Hz, 1 H, py) ppm. ^13^C NMR (150.90 MHz, 22 °C, [D_6_]DMSO): *δ*=48.0, 48.6, 52.7, 52.8, 53.3, 54.2, 63.9, 65.0, 70.5, 74.0, 74.5, 123.9, 124.1, 124.6, 125.2, 125.3, 125.9, 126.3, 140.0, 140.7, 141.6, 148.2, 148.9, 150.6, 151.1, 154.3, 154.5, 154.8, 156.4, 166.9, 168.1, 170.0, 170.2 ppm. HR‐ESI MS (pos., MeOH): *m/z* [[Bi(L^4^)]]^+^ calcd. 889.2029, obsd. 889.2054. Elemental analysis: [[Bi(L^4^)(NO_3_)]⋅4 H_2_O] calcd. (%): C 41.06, H 3.94, N 9.58; obsd. C 40.79, H 3.78, N 9.61.

*[Bi^III^(L^4’^)(NO_3_)](NO_3_)*. Bi(NO_3_)_3_⋅5 H_2_O (122 mg, 0.25 mmol, 1.0 equiv) was dissolved in 5 mL MeOH with a few drops of concentrated HNO_3_ and added to a solution of L^4^⋅H(TFA) (200 mg, 0.25 mmol, 1.0 equiv) in 10 mL MeOH. The reaction mixture was heated to 60 °C for 2 h. The solvent was evaporated and the residue recrystallized from hot MeOH (148 mg, 0.17 mmol, 67 %). ^1^H NMR (600.13 MHz, 22 °C, [D_6_]DMSO): *δ*=2.58 (d, ^2^ *J*_H,H_=12.9 Hz, 1 H, N^7^CH_2,ax/equiv)_, 3.11 (d, ^2^ *J*_H,H_=12.8 Hz, 1 H, N^7^CH_2,ax/equiv)_, 3.18 (d, ^2^ *J*_H,H_=12.8 Hz, 1 H, N^7^CH_2,ax/equiv)_, 3.27 (d, ^2^ *J*_H,H_=13.2 Hz, 1 H, N^7^CH_2,ax/equiv)_, 3.64 (s, 3 H, COOCH_3_), 3.73 (s, 3 H, COOCH_3_), 4.45 (q, ^2^ *J*_H,H_=15.1 Hz, 2 H, N^7^CH_2_pa), 4.78 (d, ^3^ *J*_H,H_=5.2 Hz, 1 H, CHOH), 5.09 (t, ^3^ *J*_H,H_=3.6 Hz, 1 H, N^3^H), 5.42 (d, ^3^ *J*_H,H_=3.6 Hz, 1 H, N^3^CH), 5.48 (d, ^3^ *J*_H,H_=3.2 Hz, 1 H, N^3^CH), 6.86 (d, ^3^ *J*_H,H_=5.9 Hz, 1 H, OH), 7.45 (d, ^3^ *J*_H,H_=7.9 Hz, 1 H, py), 7.51 (d, ^3^ *J*_H,H_=7.8 Hz, 1 H, py), 7.65‐7.68 (m, 1 H, py), 7.70–7.74 (m, 1 H, py), 8.01 (d, ^3^ *J*_H,H_=7.8 Hz, 1 H, pa), 8.10 (td, ^3^ *J*_H,H_=7.7 Hz, ^4^ *J*_H,H_=1.6 Hz, 1 H, py), 8.15 (td, ^3^ *J*_H,H_=7.7 Hz, ^4^ *J*_H,H_=1.5 Hz, 1 H, py), 8.20 (d, ^3^ *J*_H,H_=7.8 Hz, 1 H, pa), 8.44 (t, ^3^ *J*_H,H_=7.7 Hz, 1 H, pa), 8.66 (d, ^3^ *J*_H,H_=4.8 Hz, 1 H, py), 9.00 (d, ^3^ *J*_H,H_=4.8 Hz, 1 H, py) ppm. ^13^C NMR (150.90 MHz, 22 °C, [D_6_]DMSO): *δ*=48.9, 49.1, 51.8, 52.0, 52.9, 63.0, 64.3, 65.8, 71.2, 123.6, 124.9, 125.3, 126.2, 126.4, 127.5, 140.2, 141.4, 142.6, 147.4, 149.8, 150.5, 154.8, 154.9, 168.2, 169.6, 169.8 ppm. HR‐ESI MS (pos., DMSO/MeOH): *m/z* [[Bi(L^4’^)]]^+^ calcd. 754.1709, obsd. 754.1713. Elemental analysis: [[Bi(L^4’^)(NO_3_)](NO_3_)⋅H_2_O] calcd. (%): C 37.47, H 3.37, N 10.92; obsd. C 37.48, H 3.43, N 11.18.

**Radiolabeling**. ^213^Bi^III^ was eluted with a mixture of 0.2 M aqueous NaI (0.3 mL) and 0.2 M aqueous HCl (0.3 mL) as [^213^BiI_4_]^−^ and [^213^BiI_5_]^2−^ containing an activity of 11–15 MBq per elution from a ^225^Ac/^213^Bi generator system as provided by the Institute for Transuranium Elements (Karlsruhe, Germany).^3^ The eluate was adjusted to pH 5.0 with aq NH_4_OAc (3 M, 0.1 mL) and diluted with H_2_O (1 mL). Labeling was performed by addition of the buffered eluate (90 μL) to the respective ligand solution (10 μL, 0.01–300 μM) in an Eppendorf tube, resulting in final chelator concentrations of 0.001–30 μM. After incubation at 95 °C or at ambient temperature (ca. 25 °C) for 5 min, the fraction of complexed ^213^Bi^III^ was evaluated by radio‐TLC (mobile phase: aqueous sodium citrate, 0.1 M, pH 5.5).

**Transchelation challenge**. Complete labeling of the respective chelators was achieved by reacting a 10 μM ligand solution (10 μL) with 200 μL ^213^Bi^III^‐eluate at 95 °C for 5 min. The labeling solution containing the chelator radiometal complex (20 μL) was added to 0.1 M aqueous Na‐DTPA (100 μL, pH 7.5) as competing medium and incubated at 37 °C for different time periods (*t*=0, 30, 60, 120 min). The fraction of intact chelate radiometal complex was evaluated by radio‐TLC (mobile phase: aqueous sodium DTPA, 0.1 M, pH 7.5). The same experimental procedure was used for evaluation of the kinetic inertness of radiometal complexes obtained by radiometallationn reactions at 25 °C.

**log** ***D***_**7.4**_ **values**. 500 μL 1‐octanol and 500 μL phosphate buffered saline (PBS, pH 7.4) were placed in a 1.5 mL Eppendorf vial. Ca. 1 MBq of the ^213^Bi‐labeled compounds, prepared by radiolabeling at 95 °C, were added and vortexed vigorously for 3 min. Phase separation was achieved by centrifugation (13.500 g, 10 min) and the activities in 200 μL of each phase were quantified in a γ‐counter (Wizard). Values are given as averages ± standard deviations (*n*=8) of decadic logarithms of the activity contained in the octanol phase divided by that in the PBS phase.

**Supporting Information**. NMR and electronic spectra, details of the labeling studies as well as experimental details of the solid state X‐ray analyses, including the crystallographic data tables, are given as Supporting Information.

## Conflict of interest

The authors declare no conflict of interest.

## Supporting information

As a service to our authors and readers, this journal provides supporting information supplied by the authors. Such materials are peer reviewed and may be re‐organized for online delivery, but are not copy‐edited or typeset. Technical support issues arising from supporting information (other than missing files) should be addressed to the authors.

Supplementary
