# Supplementary material for: First‐Generation Bispidine Chelators for 213BiIII Radiopharmaceutical Applications
Source: ChemMedChem. 2020 Jul 2;15(16):1591–600. doi: 10.1002/cmdc.202000361 (PMC7496608; doi:10.1002/cmdc.202000361)
Supplement: Supplementary file 1 — Supplementary [file CMDC-15-1591-s001.pdf]

ChemMedChem

Supporting Information

First-Generation Bispidine Chelators for $^{213}Bi^{III}$ Radiopharmaceutical Applications

Frank Bruchertseifer, Peter Comba,* Bodo Martin, Alfred Morgenstern, Johannes Notni, Miriam Starke+, and Hubert Wadepohl

Supporting Information

1. Ligand Synthesis

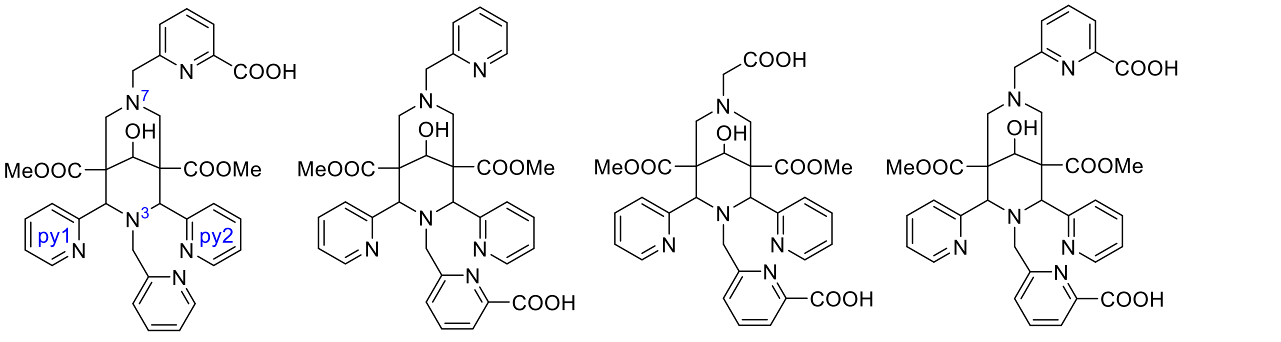

L^1 **L^2** **L^3** **L^4**

Chart S1. Ligands used.

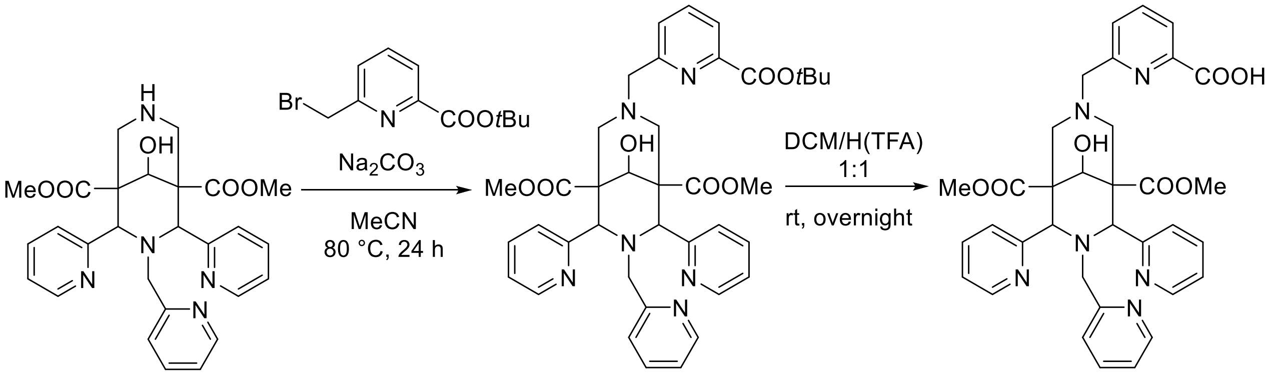

4 **5**, 95 % **L^1**, 66 %

Scheme S1. Synthesis of L^1.

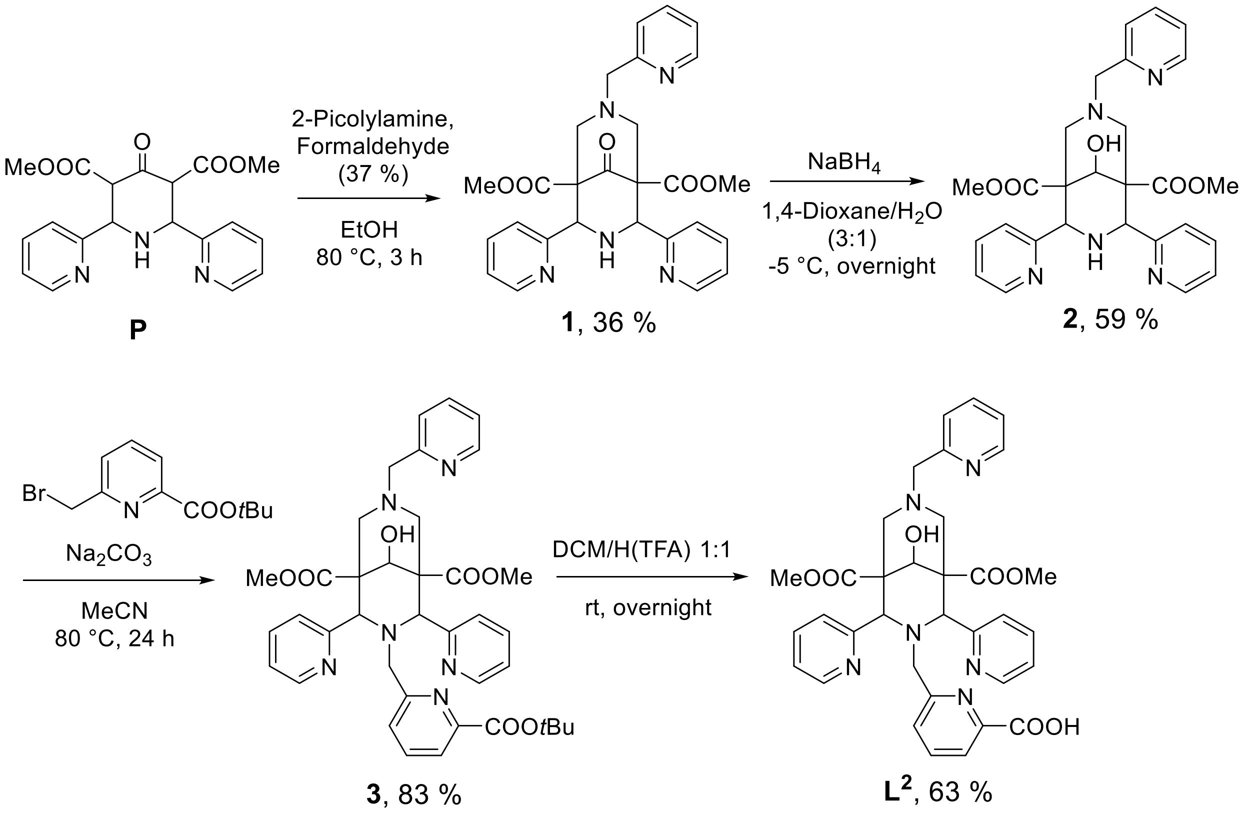

Scheme S2. Synthesis of L^2.

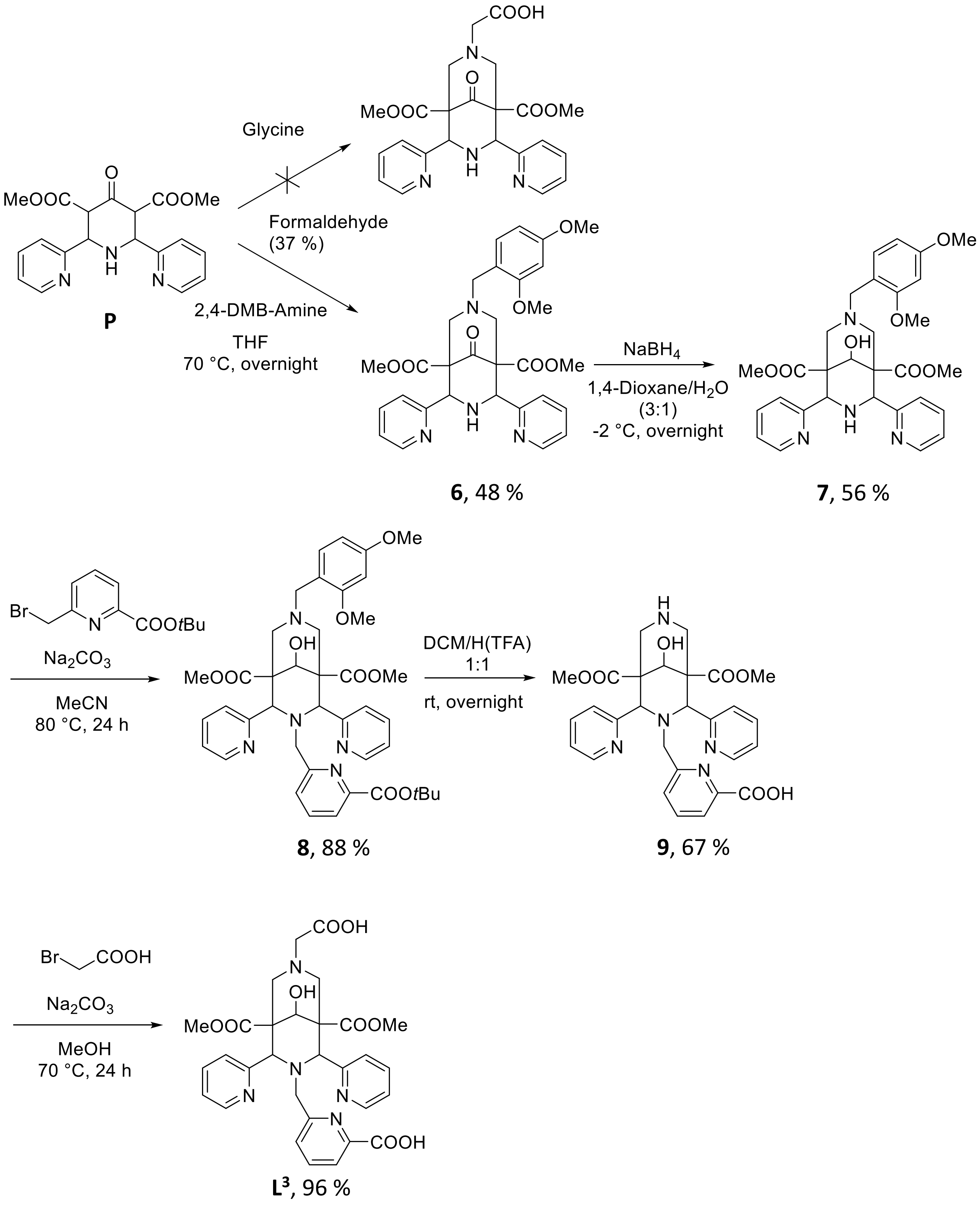

Scheme S3. Synthesis of L^3.

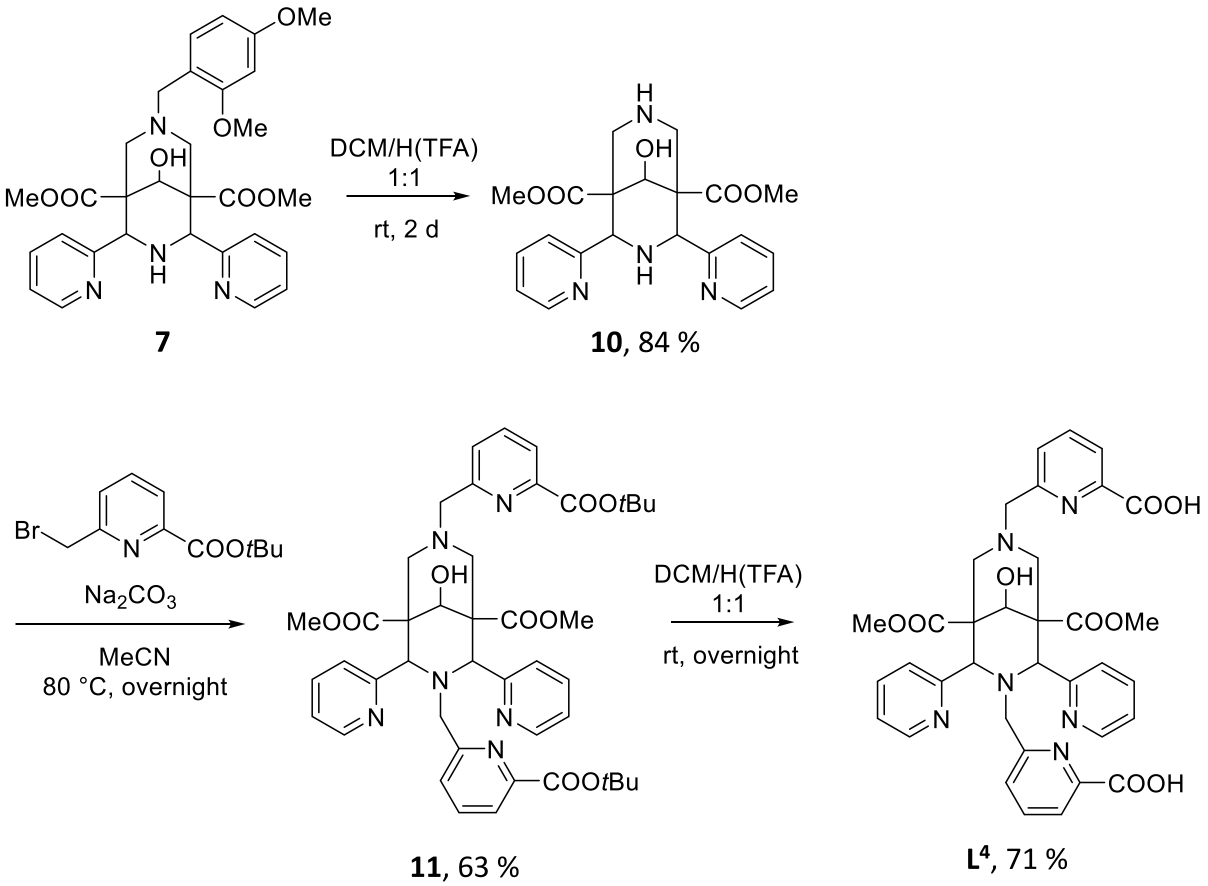

Scheme S4. Synthesis of L^{4}.[1]

1.1 NMR Spectra and X-ray Structures of Ligands and Precursors

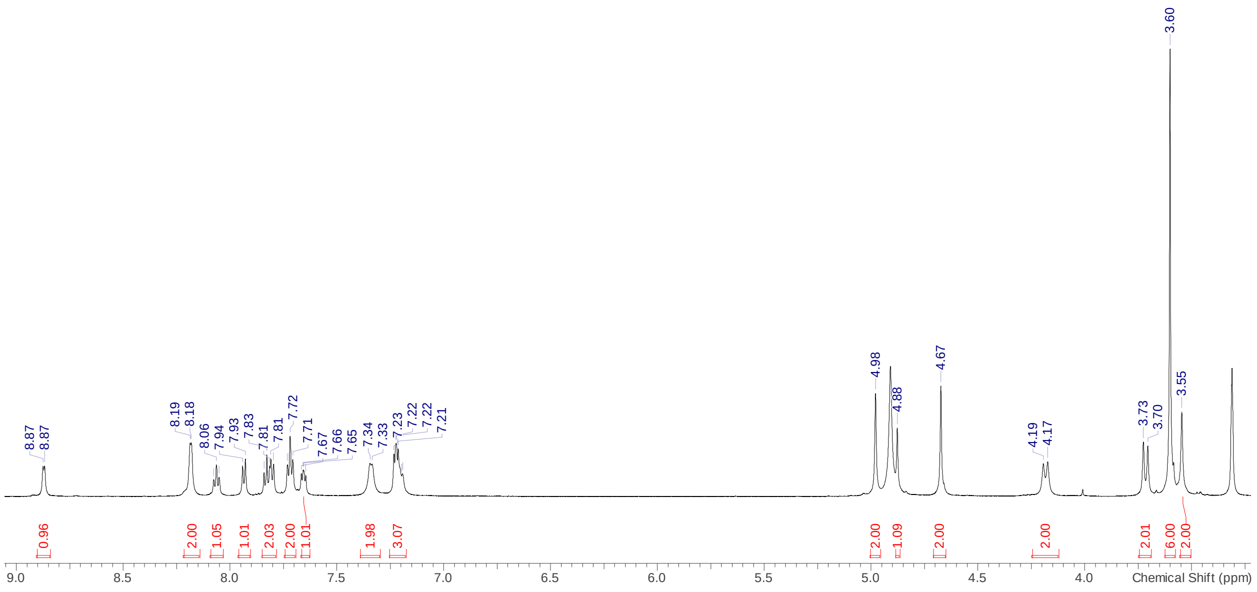

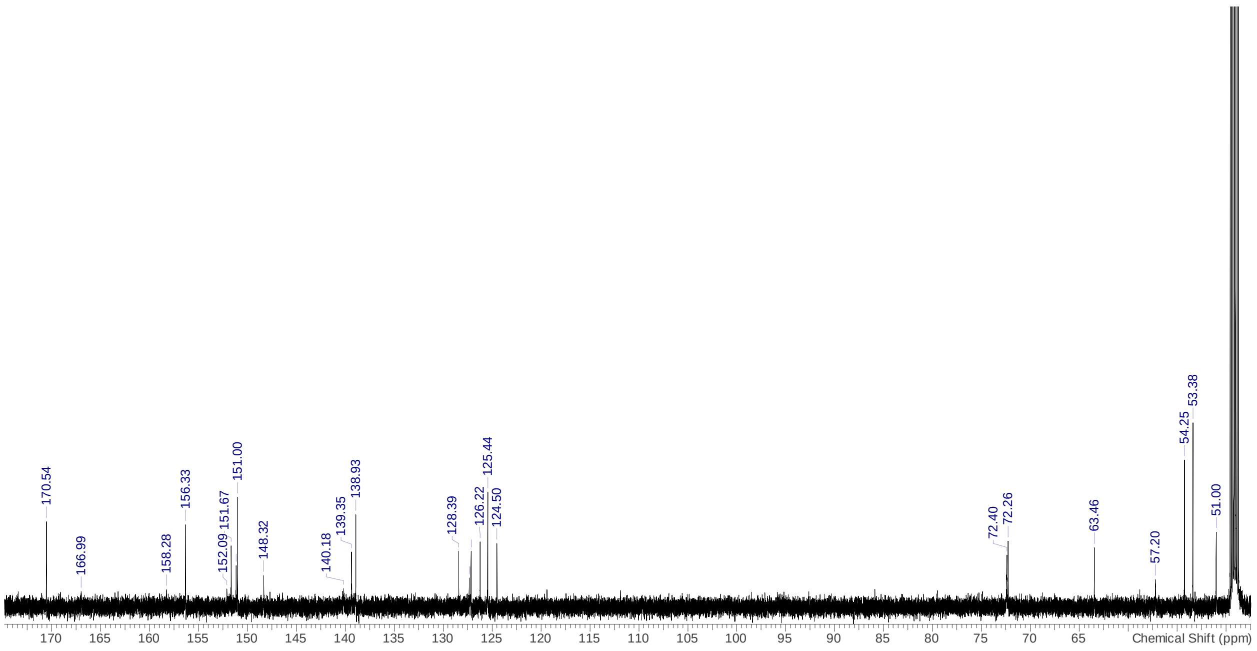

Figure S1. ^{1}H NMR (600.13 MHz, 22°C, MeOH-d4) (top) and ^{13}C NMR spectra (150.90 Hz, 22°C, MeOH-d4) (bottom) of $L^{2}\cdot H(TFA)$.

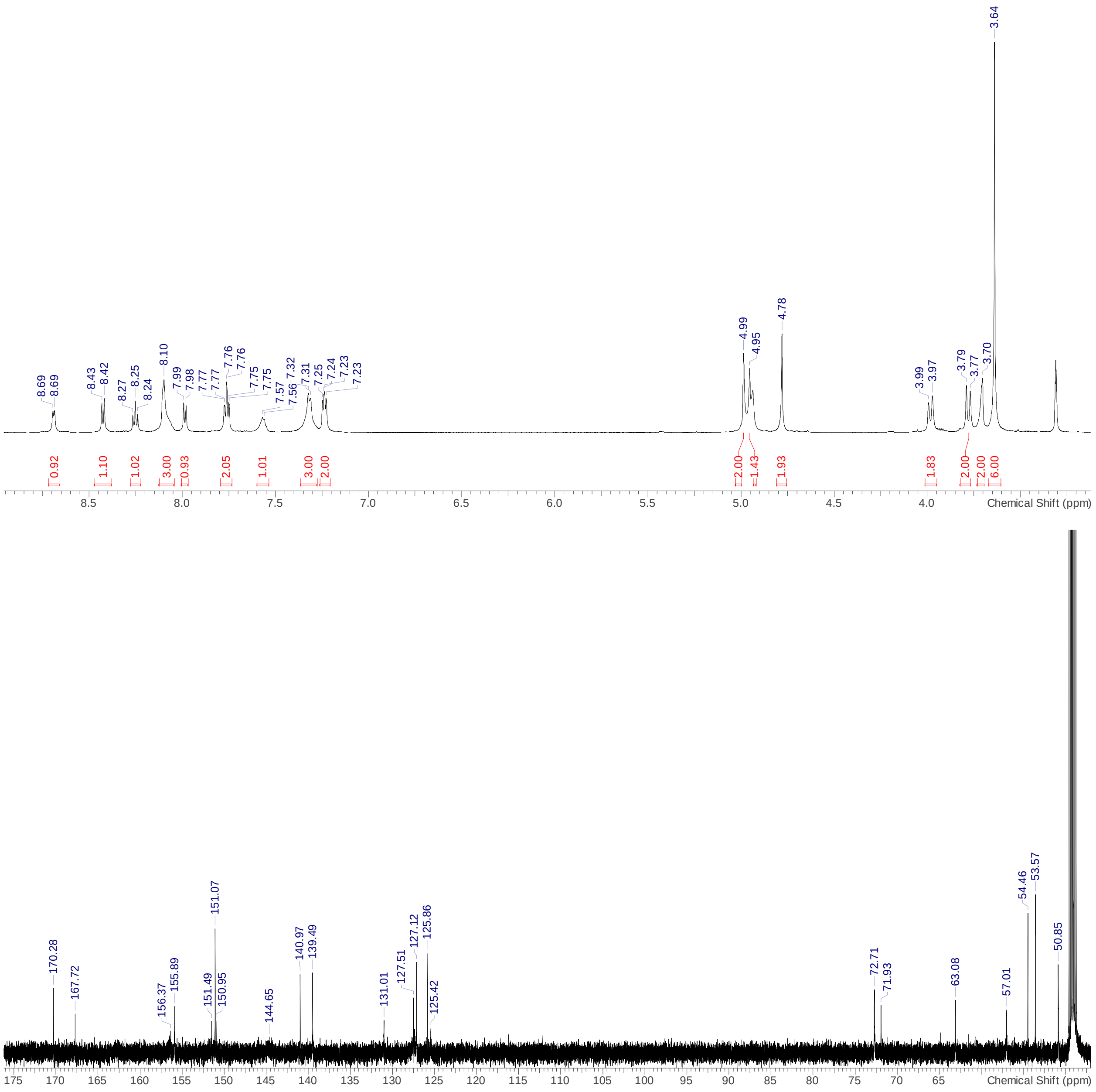

Figure S2. ^{1}H NMR (600.13 MHz, 22°C, MeOH-d$_4$) (top) and ^{13}C NMR spectra (150.90 Hz, 22°C, MeOH-d$_4$) (bottom) of L^1·2 H(TFA).

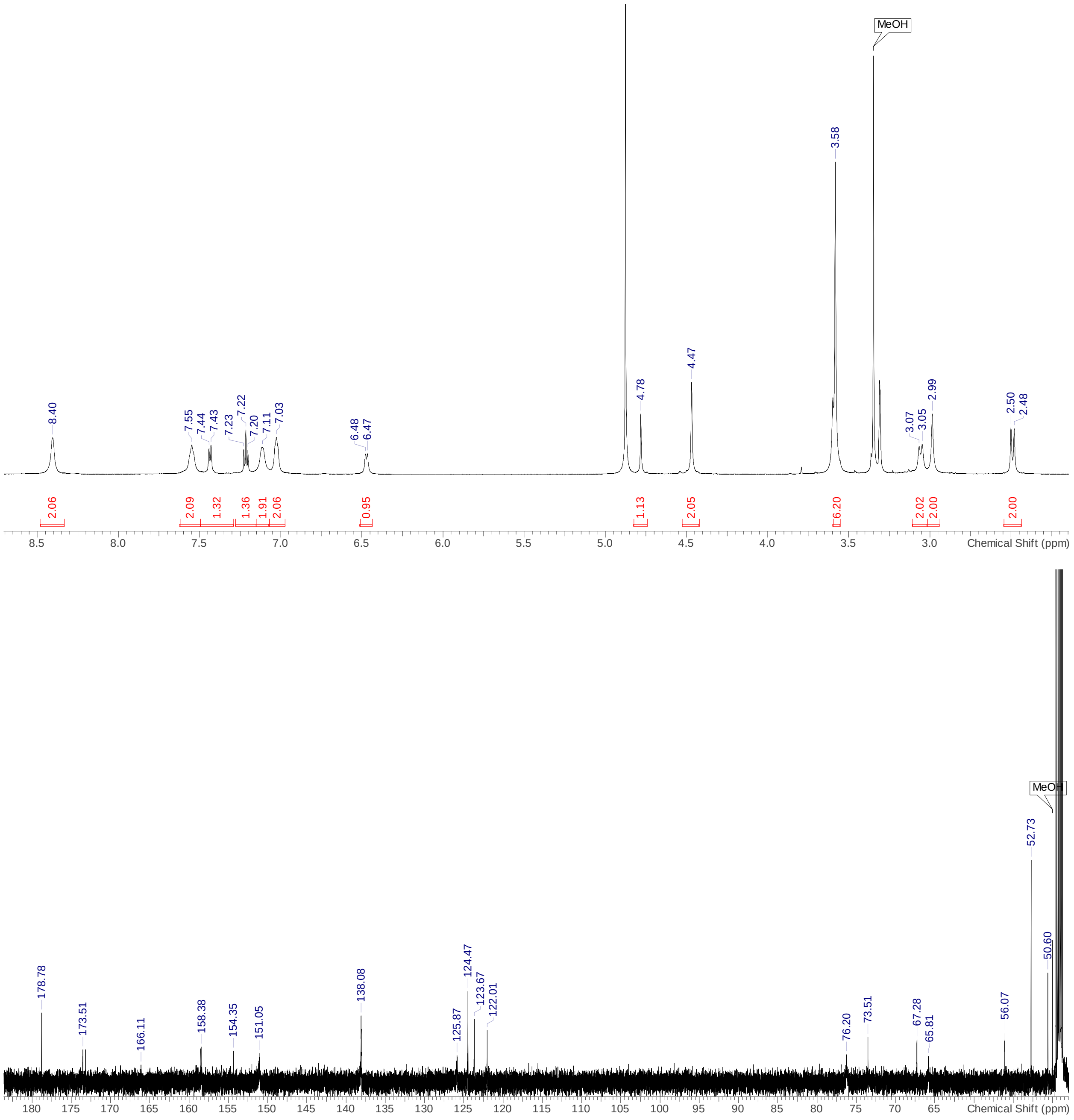

Figure S3. ^{1}H NMR (600.13 MHz, 22°C, MeOH-d_4) (top) and ^{13}C-NMR spectra (150.90 Hz, 22°C, MeOH-d_4) (bottom) of $L^3 \cdot Na_2CO_3 \cdot NaBr$.

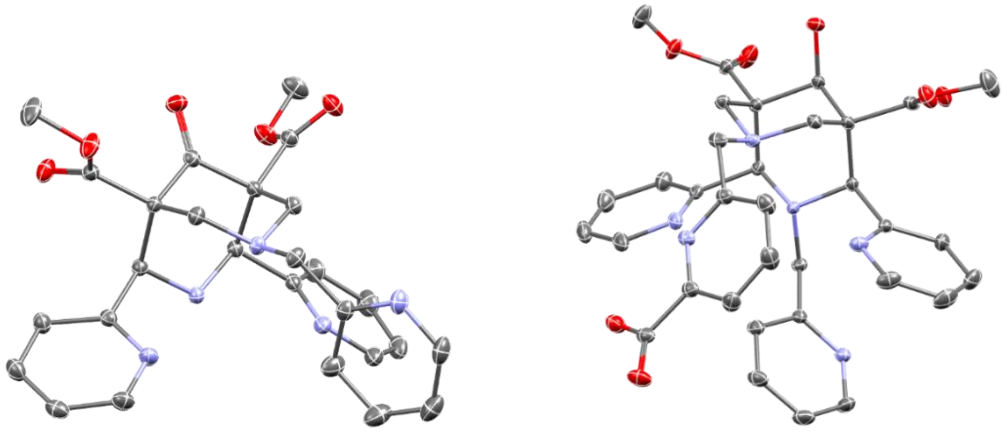

Figure S4. Adp plots of the single crystal X-ray structural analysis of **1** (left) and $[L^1\cdot 2\ H]^{2+}$ (right). Hydrogen atoms, triflate anions and solvent molecules are omitted for clarity. Color code, C: grey, N: blue, O: red.

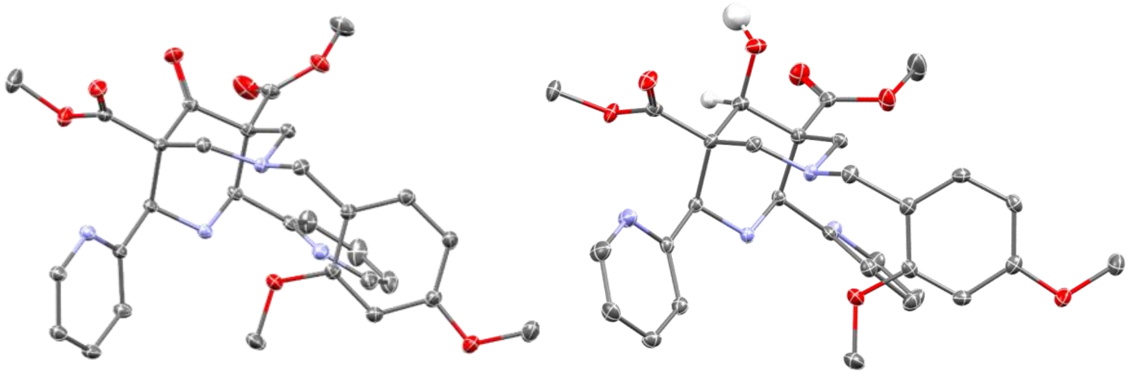

Figure S5. Adp plots of the single crystal X-ray structural analysis of **6** (left) und **7** (right; only one of the two independent hydrogen-bridged molecules is shown; note that the plot of **6** has been inflected to have similar orientations of the two molecules). Hydrogen atoms and solvent molecules are omitted for clarity, except for the hydrogen atoms at C9 and the corresponding alcohol group to show the difference between the ligands **6** and **7**. Color code, C: grey, N: blue, O: red.

2. NMR Spectroscopy and Crystal Structures of the Bi^{III} Complexes

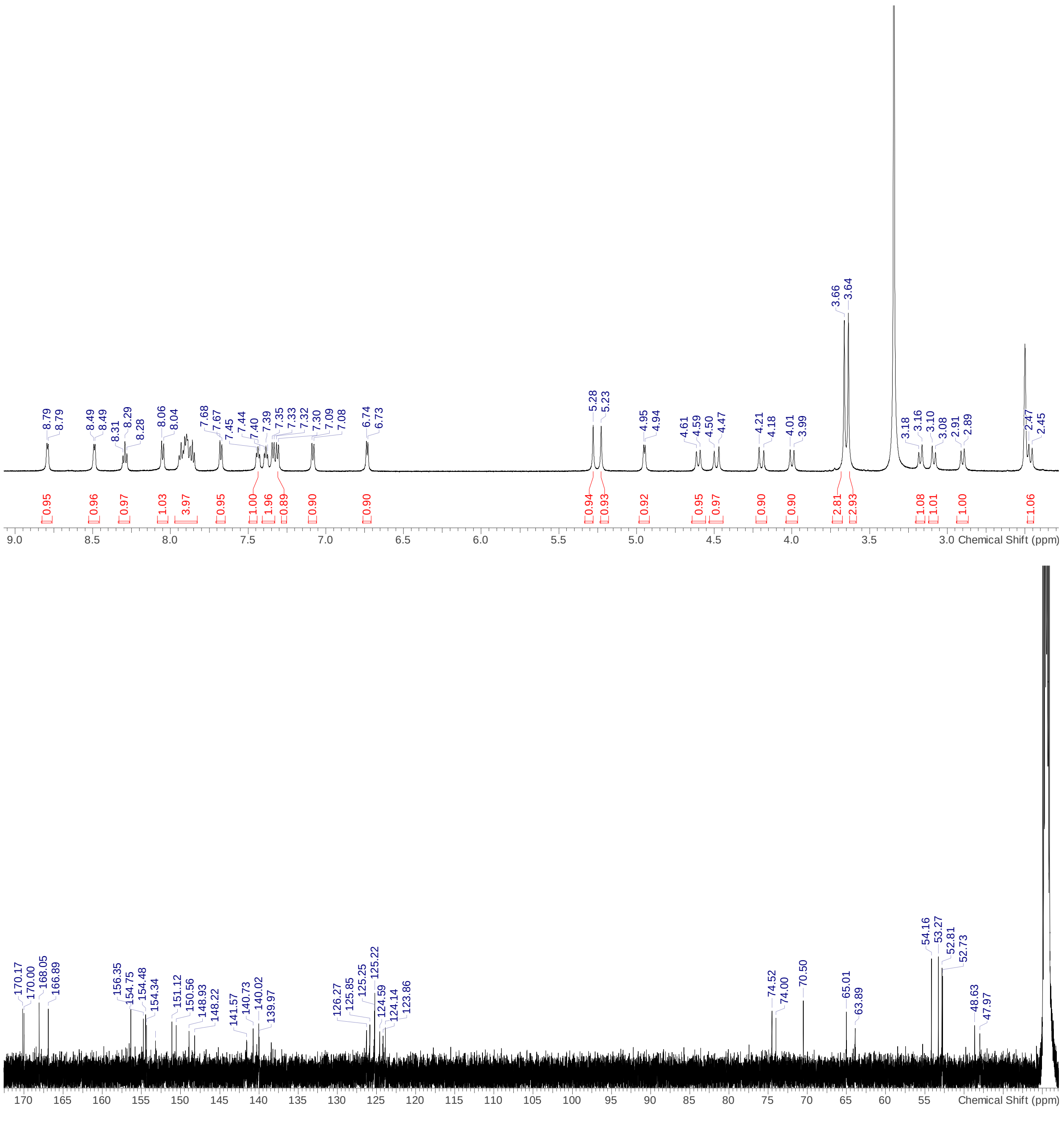

Figure S6.^{1}H NMR (600.13 MHz, 22°C, DMSO-d_6) (top) and ^{13}C NMR spectra (150.90 Hz, 22°C, DMSO-d_6) (bottom) of [Bi^{III}(L^4)(NO_3)].

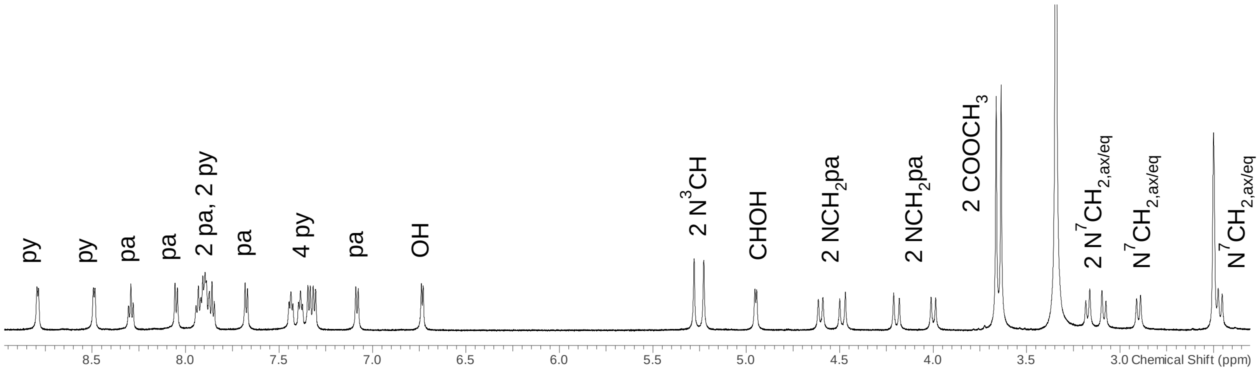

Figure S7. ^{1}H NMR spectrum of $[Bi^{III}(L^4)(NO_3)]$ in DMSO-d_6.

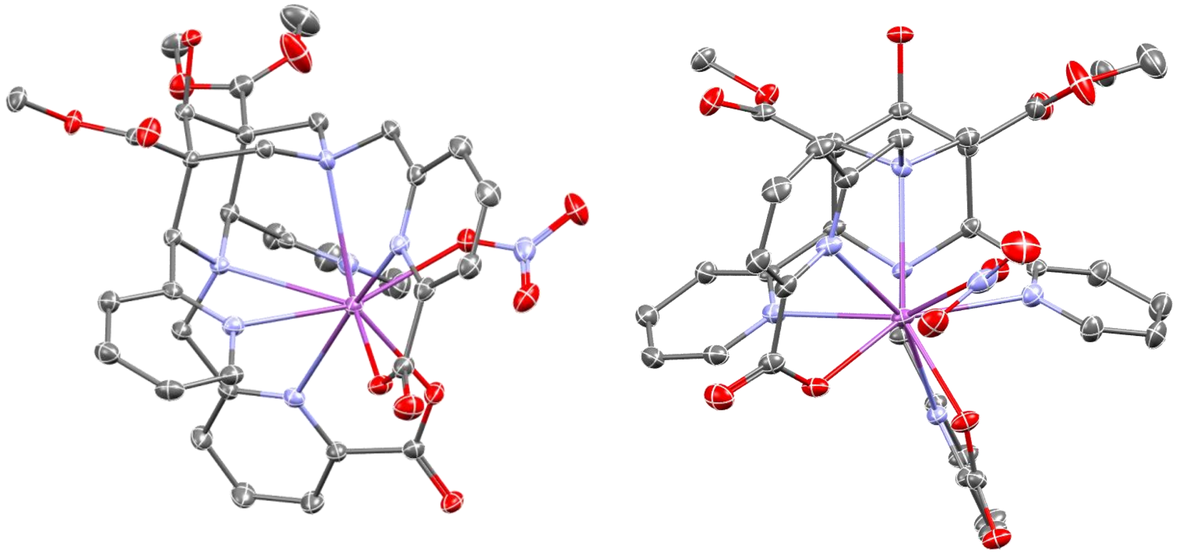

Figure S8. Adp plots of two views of the single crystal X-ray structural analysis of $[Bi^{III}(L^4)(NO_3)]$. Hydrogen atoms are omitted for clarity. Color code, C: grey, N: blue, O: red, Bi: violet.

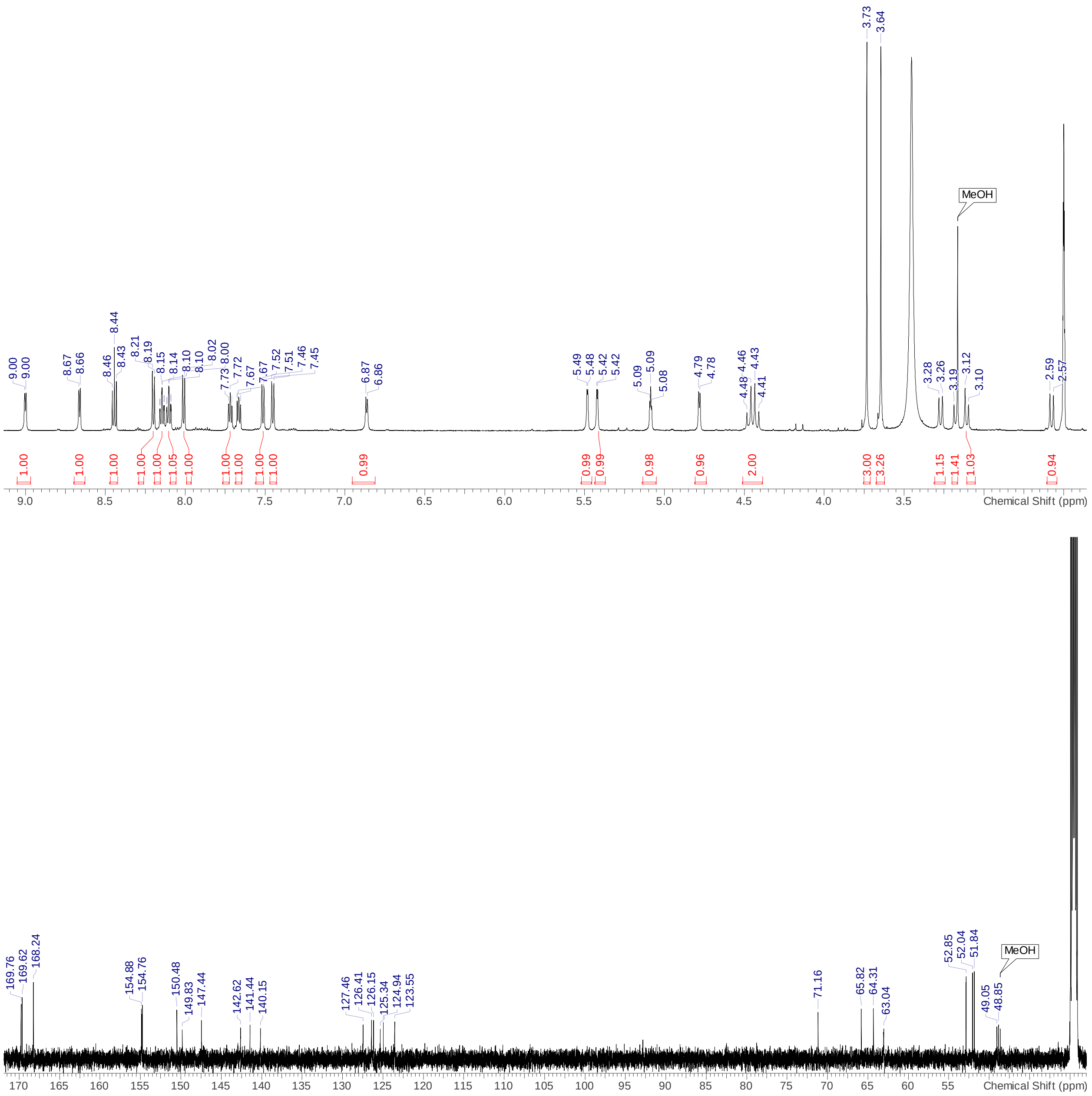

Figure S9.^{1}H NMR (600.13 MHz, 22°C, DMSO-d$_6$) (top) and ^{13}C NMR (150.90 Hz, 22°C, DMSO-d$_6$) spectra (bottom) of [Bi^{III}(L$^{4'}$)(NO$_3$)](NO$_3$).

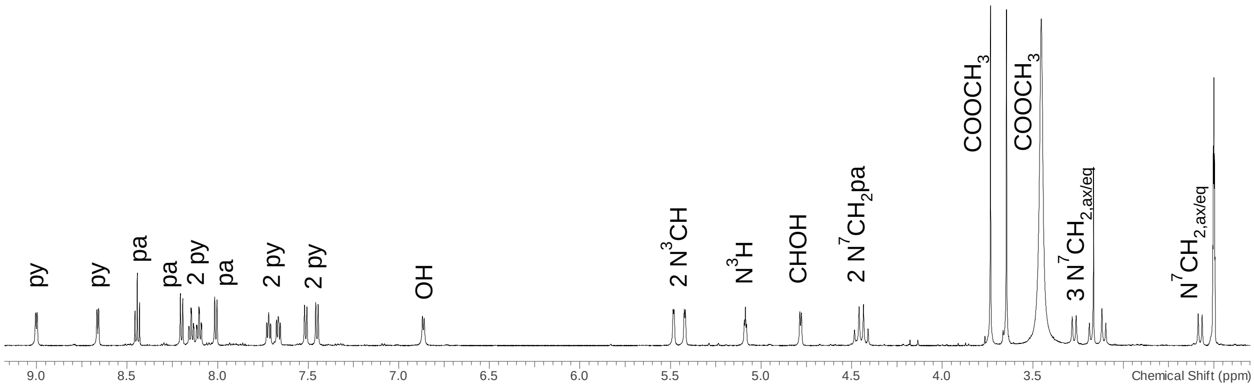

Figure S10.^{1}H NMR spectrum of [Bi^{III}(L$^{4'}$)(NO_3)](NO_3) in DMSO-d_6.

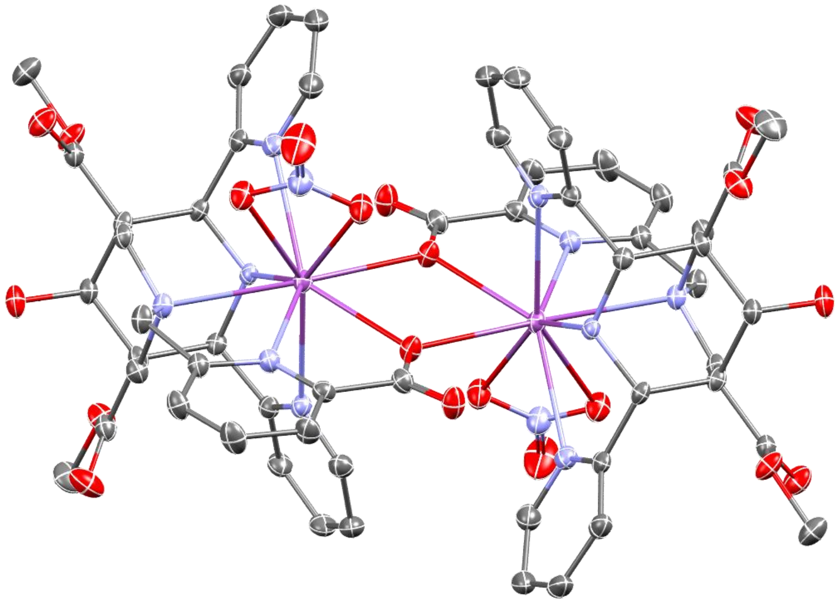

Figure S11. Adp plot of the single crystal X-ray structural analysis of {[Bi^{III}(L$^{4'}$)(NO_3)]$_2$}$^{2+}$. Hydrogen atoms are omitted for clarity. Color code, C: grey, N: blue, O: red, Bi: violet.

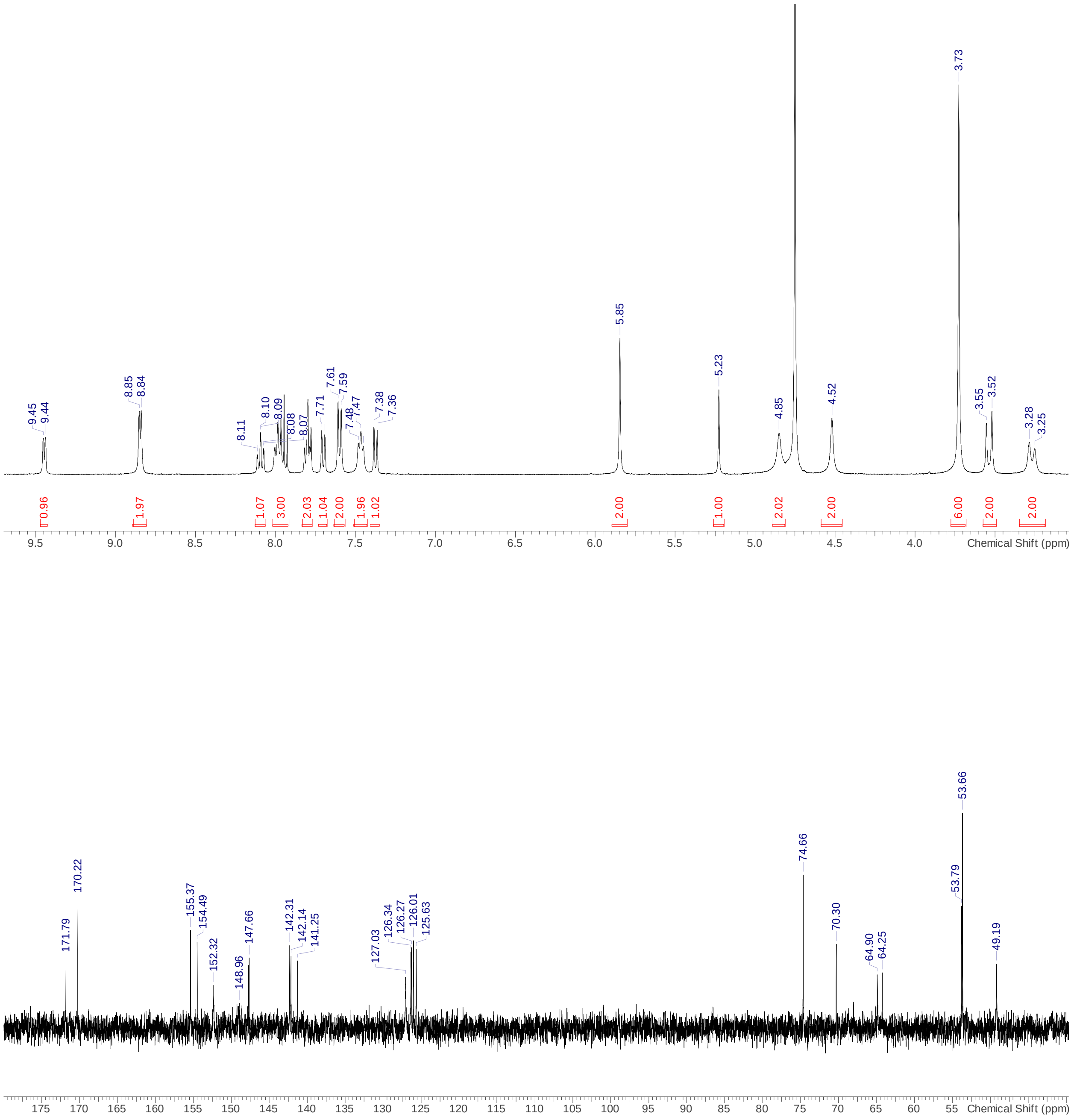

Figure S12.^{1}H NMR (399.89 MHz, 22°C, D_2O) (top) and ^{13}C NMR spectra (100.55 Hz, 22°C, D_2O) (bottom) of $[Bi^{III}(L^2)](NO_3)_2$.

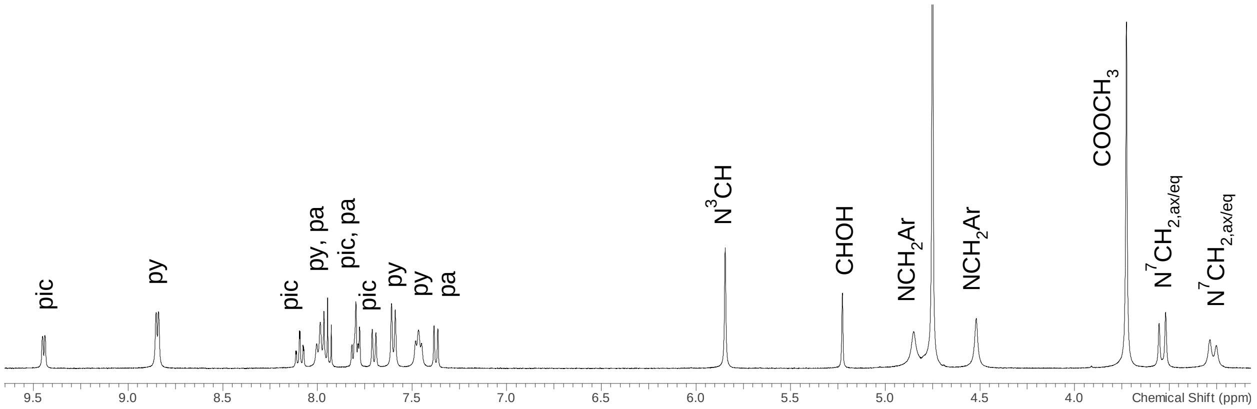

Figure S13. ^{1}H NMR spectrum of $[Bi^{III}(L^2)](NO_3)_2$ in D_2O.

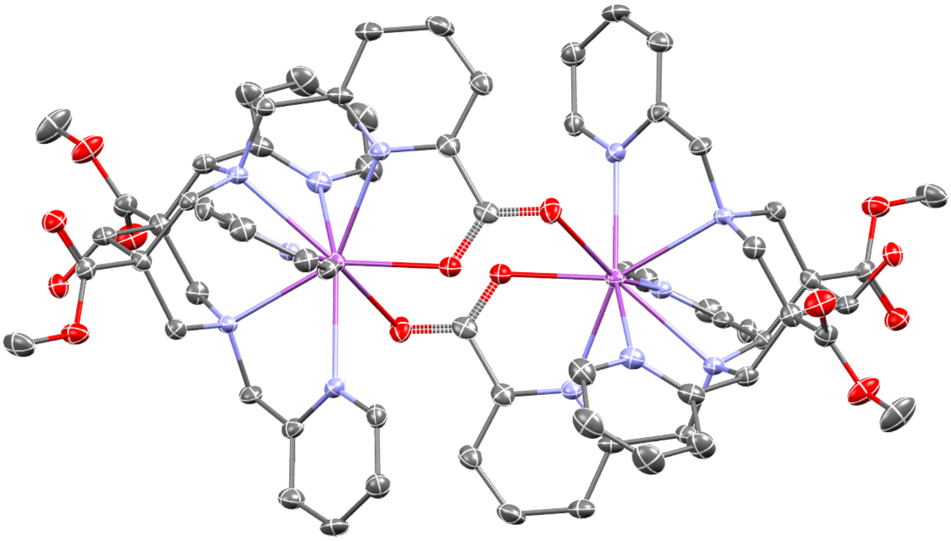

Figure S14. Adp plot of the single crystal X-ray structural analysis of $\{[Bi^{III}(L^2)]_2\}^{4+}$. Hydrogen atoms are omitted for clarity. Color code, C: grey, N: blue, O: red, Bi: violet.

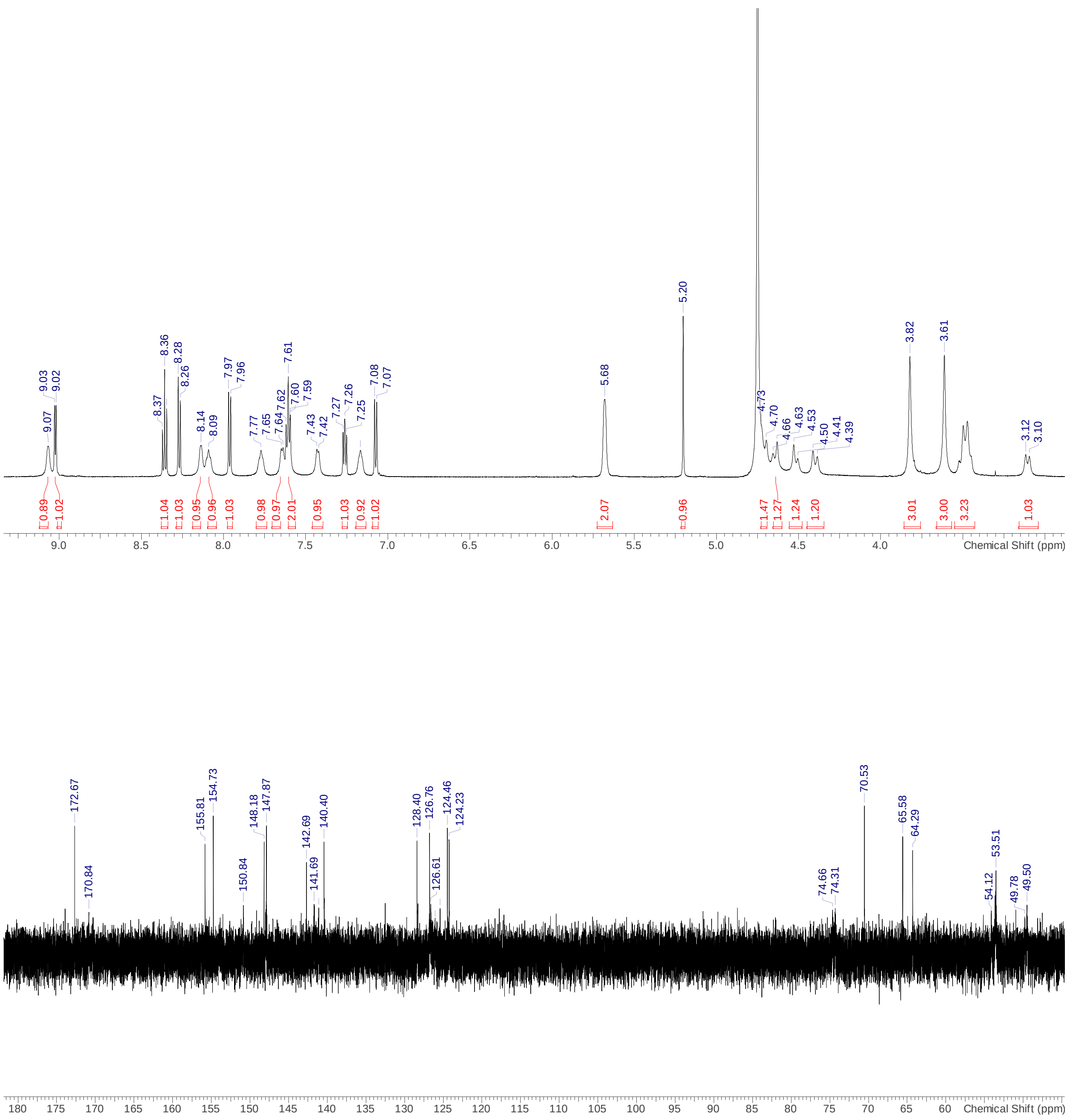

Figure S15. ^{1}H NMR (600.13 MHz, 22°C, D_2O) (top) and ^{13}C NMR spectra (150.90 Hz, 22°C, D_2O) (bottom) of $[Bi^{III}(L^1)(NO_3)](NO_3)$.

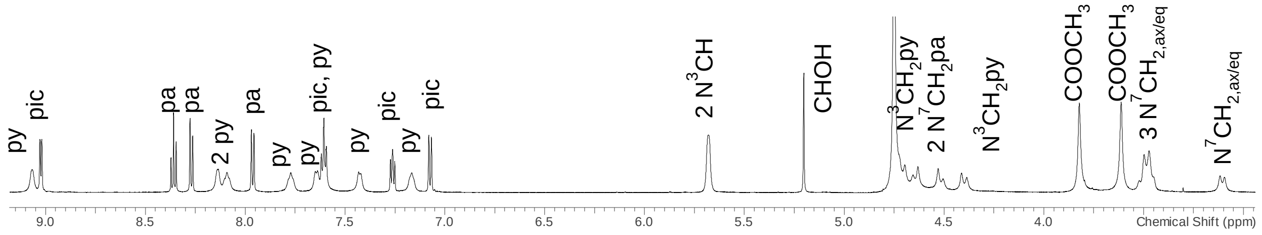

Figure S16. ^{1}H NMR spectrum of $[Bi^{III}(L^1)(NO_3)](NO_3)$ in D_2O.

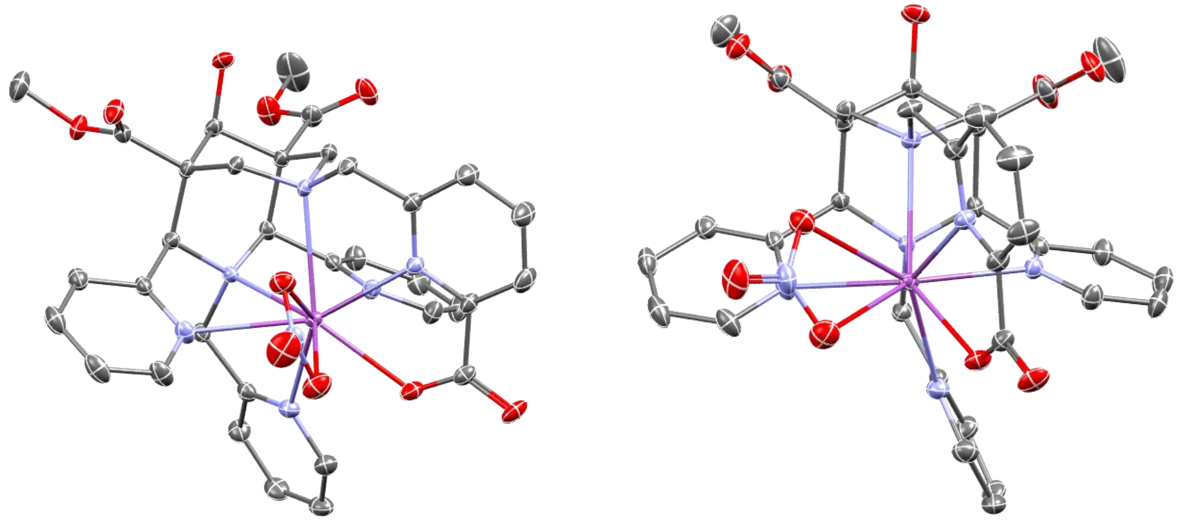

Figure S17. Adp plots of two views of the single crystal X-ray structural analysis of $[Bi^{III}(L^1)(NO_3)]^+$. Hydrogen atoms are omitted for clarity. Color code, C: grey, N: blue, O: red, Bi: violet.

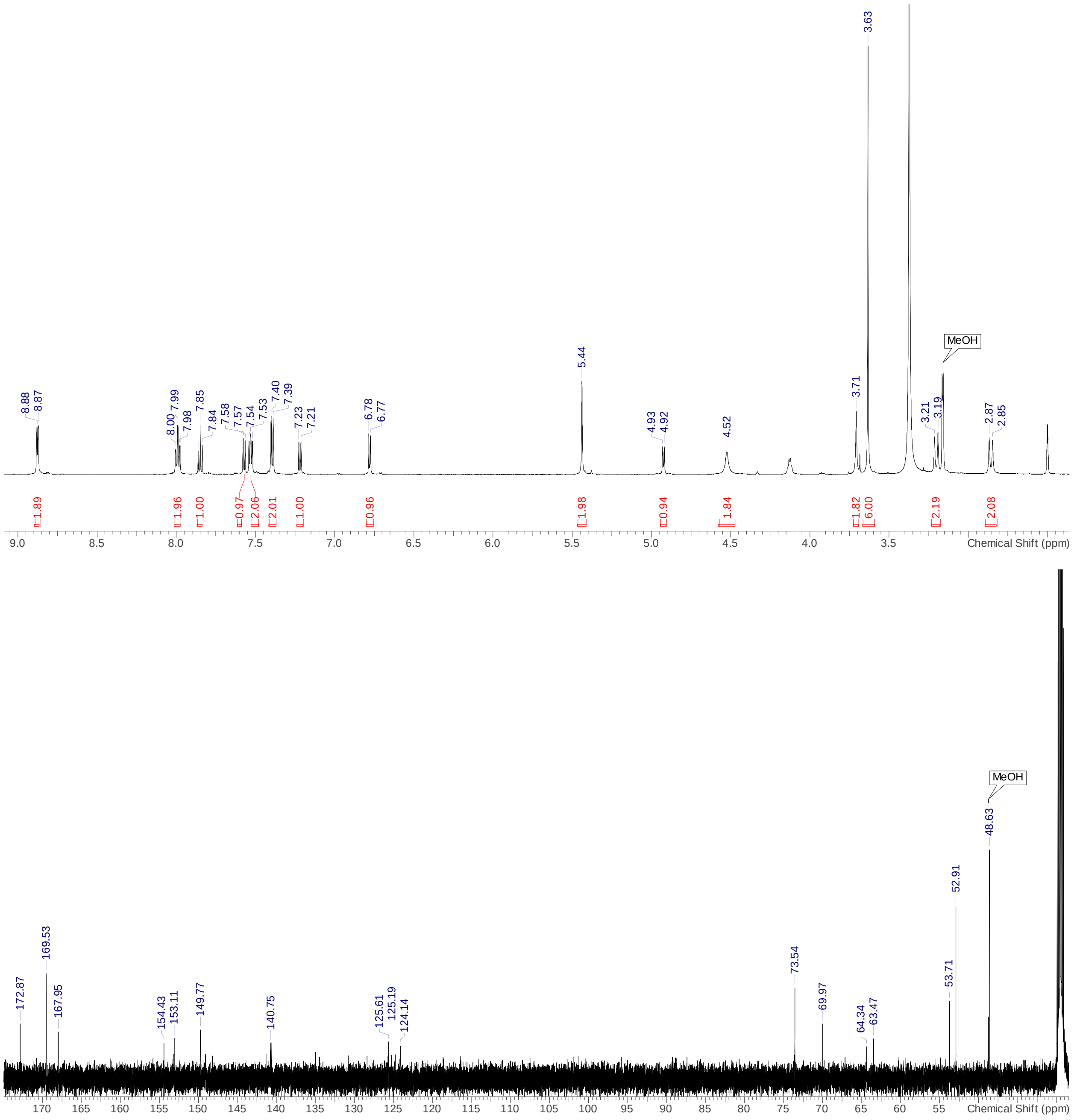

Figure S18. ^{1}H NMR (600.13 MHz, 22°C, DMSO-d$_6$) (top) and ^{13}C NMR spectra (150.90 Hz, 22 °C, DMSO-d$_6$) (bottom) of $[Bi^{III}(L^3)(Br)_x(NO_3)_{1-x}]$.

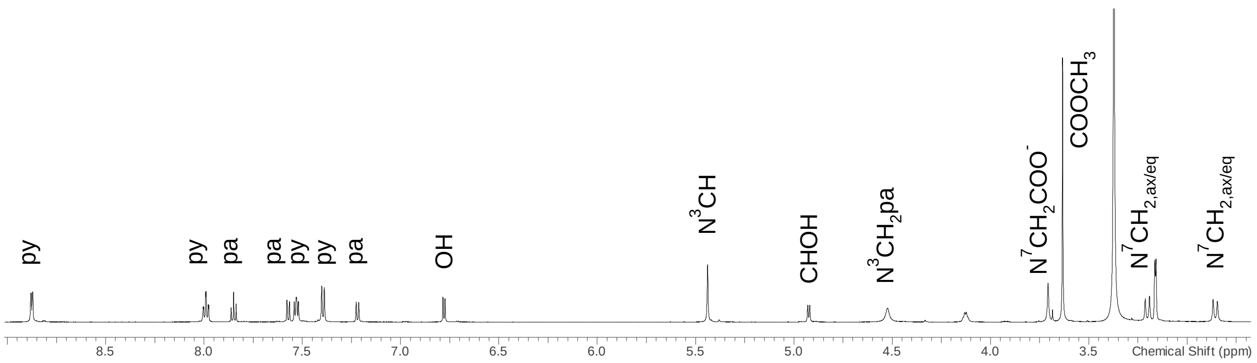

Figure S19. ^{1}H NMR spectrum of $[Bi^{III}(L^3)(Br)_x(NO_3)_{1-x}]$ in DMSO-d$_6$.

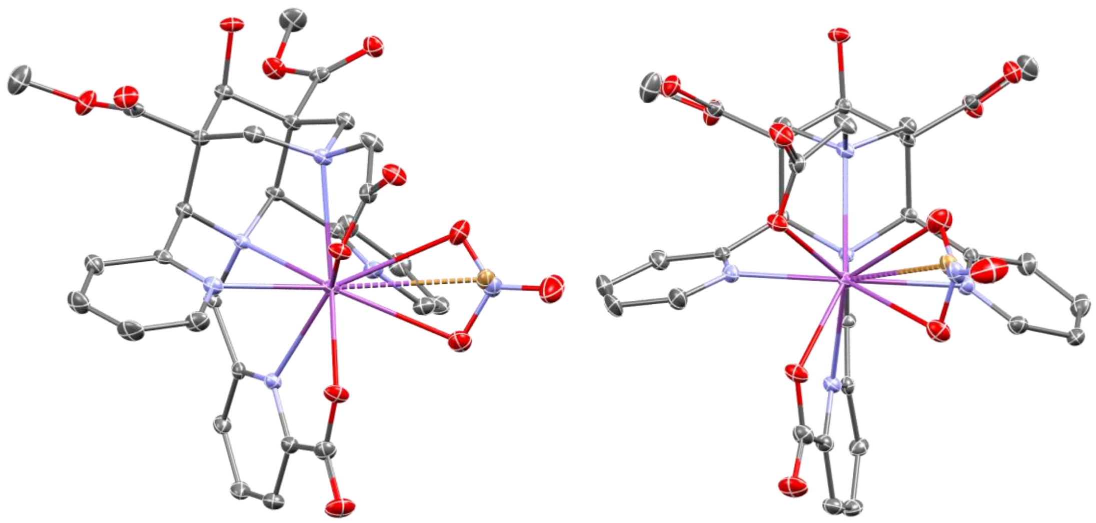

Figure S20. Adp plots of two views of the single crystal X-ray structural analysis of $[Bi^{III}(L^3)(Br)_{0.6}(NO_3)_{0.4}]$ (plotted is the structure with coordinated nitrate). Hydrogen atoms are omitted for clarity. Color code, C: grey, N: blue, O: red, Bi: violet.

3. Crystal Structural Analyses

Crystal data and details of the structure determinations are compiled in Tables S1. Full shells of intensity data were collected at low temperature with a Bruker AXS Smart 1000 CCD diffractometer (Mo-K_a radiation, sealed X-ray tube, graphite monochromator; compounds **L^1**·2 H(TFA)·2 iPrOH, **6**, **7**, $[Bi^{III}(L^{4'})(NO_3)]_2(NO_3)_2$ and $[Bi^{III}(L^3)(Br)_{0.6}(NO_3)_{0.4}]$·MeOH) or an Agilent Technologies Supernova-E CCD diffractometer (Mo-K_a radiation, microfocus X-ray tube, multilayer mirror optics; all other compounds). Detector frames (typically w-, occasionally j-scans, scan width 0.4 … 0.5°) were integrated by profile fitting.[2-5] Data were corrected for air and detector absorption, Lorentz and polarization effects[3-5] and scaled essentially by application of appropriate spherical harmonic functions.[5-9] Absorption by the crystal was treated with a semiempirical multiscan method (as part of the scaling process), and augmented by a spherical correction,[5-9] or numerically (Gaussian grid).[5, 9, 10] For datasets collected with the microfocus tube an illumination correction was performed as part of the numerical absorption correction.[5, 9]

The structures were solved by intrinsic phasing[11-13] (complex $[Bi^{III}(L^3)(Br)_{0.6}(NO_3)_{0.4}]$·MeOH), by the heavy atom method combined with structure expansion by direct methods applied to difference structure factors[14, 15] (complex $[Bi^{III}(L^4)(NO_3)]\cdot x(H_2O, MeOH)$), by ab initio dual space methods involving difference Fourier syntheses[16, 17] (VLD procedure, compounds 7 and $[Bi^{III}(L^2)]_2(NO_3)_4$·4 MeOH) or by the charge flip procedure[18, 19] (all other compounds) and refined by full-matrix least squares methods based on F^2 against all unique reflections.[13, 20-22] All non-hydrogen atoms were given anisotropic displacement parameters. Hydrogen atoms were generally input at calculated positions and refined with a riding model.[23-26] When justified by the quality of the data the positions of some hydrogen atoms (typically those of the OH and NH groups) were taken from difference Fourier syntheses and refined. When found necessary, suitable geometry and adp restraints were applied to disordered parts of a structure.[23-26]

Due to severe disorder, electron density attributed to solvent of crystallization was removed from the structures of $[Bi^{III}(L^4)(NO_3)]$ (methanol, water), $[Bi^{III}(L^2)]_2(NO_3)_4$ (methanol?) and $[Bi^{III}(L^1)(NO_3)](NO_3)$ (methanol and/or water) with the BYPASS procedure,[27, 28] as implemented in PLATON (squeeze/hybrid).[29, 30] Partial structure factors from the solvent masks were included in the refinement as separate contributions to F_{calc}.

CCDC 1916434 - 1916442 contains the supplementary crystallographic data for this paper. These data can be obtained free of charge from the Cambridge Crystallographic Data Centre's and FIZ Karlsruhe's joint Access Service via https://www.ccdc.cam.ac.uk/structures/?.

Table S1. Crystallographic Data Table

	CCDC 1916434	CCDC 1916435
Identification code	**co_mkn11**	**co_mkn24**
Empirical formula	**1**·H_2O<br>$C_{27}H_{29}N_5O_6$	**L^1**·2 H(TFA)·2 iPrOH<br>$C_{44}H_{52}F_6N_6O_{13}$
Formula weight	519.55	986.91
Temperature	120(1) K	100(1) K
Wavelength	Mo K_α, 0.71073 Å	Mo K_α, 0.71073 Å
Crystal system	monoclinic	triclinic
Space group	$P\ 2_1/n$ (*IT* Nr. 14)	*P* -1 (*IT* Nr. 2)
Space group (Hall symbol)	-P 2yn	-P 1
Unit cell dimensions	a = 11.46815(17) Å	a = 12.158(7) Å
	b = 16.70278(19) Å	b = 13.967(7) Å
	c = 13.9810(2) Å	c = 15.289(8) Å
	α = 90 °	α = 66.465(13) °
	β = 110.9754(16) °	β = 78.185(14) °
	γ = 90 °	γ = 77.684(9) °
Volume	2500.60(6) Å^3	2305(2) Å^3
Z	4	2
Density (calculated)	1.380 Mg·m^{-3}	1.422 Mg·m^{-3}
Absorption coefficient	0.099 mm^{-1}	0.120 mm^{-1}
F_{000}	1096	1032
Crystal size	0.238 · 0.206 · 0.126 mm^3 (irregular)	0.270 · 0.230 · 0.075 mm^3 (block)
θ range for data collection	2.860 to 32.419 °	1.608 to 32.500 °
Reflections collected	104202	59277
Independent reflections [R_{int}]	8760 [0.0391]	15486 [0.0408]
Observed reflections [$I > 2\sigma(I)$]	7464	10739
Index ranges *h, k, l*	-16 ... 17, -24 ... 25, -21 ... 21	-18 ... 18, -20 ... 20, -22 ... 23
Completeness to θ = 25.242 °	99.90%	100.00%
Absorption correction	Gaussian	Semi-empirical from equivalents
Transmission factors: max, min	0.990, 0.983	0.7464, 0.6874
Structure solution[(a)]	iterative	iterative
Refinement method	Full-matrix least-squares on F^2 [(b)]	Full-matrix least-squares on F^2 [(c)]
Data / restraints / parameters	8760 / 0 / 357	15486 / 39 / 652
Goodness-of-fit on F^2	1.04	1.027
Final *R* indices [$F_o > 4\sigma(F_o)$] $R(F)$, $wR(F^2)$	0.0418, 0.1035	0.0594, 0.1489
Final *R* indices (all data) $R(F)$, $wR(F^2)$	0.0506, 0.1082	0.0927, 0.1690
Difference density: rms, max, min	0.049, 0.458, -0.206 e·Å^{-3}	0.074, 0.849, -0.698 e·Å^{-3}
Diffractometer	Agilent SuperNova	Bruker AXS Smart 1000

(a) SUPERFLIP (Palatinus, 2007-2009); (b) ShelXL (Sheldrick, 2015) Vers. 2016/6; (c) ShelXL (Sheldrick, 2015) Vers. 2018/3.

	CCDC 1916436	CCDC 1916437
Identification code	**co_mkn18**	**co_mkn21a**
Empirical formula	**6**<br>$C_{30}H_{32}N_4O_7$	**7**<br>$C_{30}H_{34}N_4O_7$
Formula weight	560.59	562.61
Temperature	100(1) K	100(1) K
Wavelength	Mo K_α, 0.71073 Å	Mo K_α, 0.71073 Å
Crystal system	monoclinic	monoclinic
Space group	$P\ 2_1/n$ (*IT* Nr. 14)	$P\ 2_1/c$ (*IT* Nr. 14)
Space group (Hall symbol)	-P 2yn	-P 2ybc
Unit cell dimensions	a = 13.058(7) Å	a = 11.569(7) Å
	b = 14.504(8) Å	b = 32.161(17) Å
	c = 14.696(8) Å	c = 14.877(9) Å
	α = 90 °	α = 90 °
	β = 104.587(14) °	β = 90.009(13) °
	γ = 90 °	γ = 90 °
Volume	2694(3) $Å^3$	5535(5) $Å^3$
Z	4	8
Density (calculated)	1.382 $Mg \cdot m^{-3}$	1.350 $Mg \cdot m^{-3}$
Absorption coefficient	0.100 mm^{-1}	0.097 mm^{-1}
F_{000}	1184	2384
Crystal size	0.430 · 0.340 · 0.280 mm^3 (block)	0.200 · 0.130 · 0.010 mm^3 (plate)
θ range for data collection	1.867 to 32.429 °	1.508 to 26.453 °
Reflections collected	67496	104627
Independent reflections [R_{int}]	9224 [0.0324]	11384 [0.0804]
Observed reflections [$I > 2\sigma(I)$]	7769	8220
Index ranges *h, k, l*	-19 ... 19, -20 ... 21, -22 ... 21	-14 ... 14, -40 ... 40, -18 ... 18
Completeness to θ = 25.242 °	100.00%	100.00%
Absorption correction	Semi-empirical from equivalents	Semi-empirical from equivalents
Transmission factors: max, min	0.8623, 0.7956	0.8614, 0.8248
Structure solution	iterative[(a)]	dual[(b)]
Refinement method[(c)]	Full-matrix least-squares on F^2	Full-matrix least-squares on F^2
Data / restraints / parameters	9224 / 0 / 439	11384 / 0 / 891
Goodness-of-fit on F^2	1.033	1.022
Final *R* indices [$F_o > 4\sigma(F_o)$] $R(F)$, $wR(F^2)$	0.0432, 0.1136	0.0419, 0.0827
Final *R* indices (all data) $R(F)$, $wR(F^2)$	0.0531, 0.1232	0.0706, 0.0942
Difference density: rms, max, min	0.064, 0.584, -0.222 $e \cdot Å^{-3}$	0.048, 0.319, -0.234 $e \cdot Å^{-3}$
Diffractometer	Bruker AXS Smart 1000	Bruker AXS Smart 1000

[(a)] SUPERFLIP (Palatinus, 2007-2009); [(b)] SIR2014 (Giacovazzo et al., 2014); [(c)] ShelXL (Sheldrick, 2015) Vers. 2018/3.

	CCDC 1916438	CCDC 1916439	CCDC 1916440
Identification code	**co_mkn26a_sq**	**co_mkn17**	**co_mkn25_sq**
Empirical formula	**[Bi^{III}(L^4)(NO_3)]**·H_2O $C_{35}H_{34}BiN_7O_{13}$	**[Bi^{III}($L^{4'}$)(NO_3)]$_2$(NO_3)$_2$** $C_{56}H_{56}Bi_2N_{14}O_{26}$	**[Bi^{III}(L^2)]$_2$(NO_3)$_4$·**4 MeOH $C_{72}H_{82}Bi_2N_{16}O_{30}$
Formula weight	969.67	1759.1	2069.49
Temperature	120(1) K	100(1) K	120(1) K
Wavelength	Mo K_α, 0.71073 Å	Mo K_a, 0.71073 Å	Mo K_α, 0.71073 Å
Crystal system	monoclinic	monoclinic	monoclinic
Space group	$P\ 2_1/c$ (*IT* Nr. 14)	$P\ 2_1/c$ (*IT* Nr. 14)	$P\ 2_1/c$ (*IT* Nr. 14)
Space group (Hall symbol)	-P 2ybc	-P 2ybc	-P 2ybc
Unit cell dimensions	a = 14.2458(4) Å	a = 11.743(8) Å	a = 12.7622(3) Å
	b = 13.85883(16) Å	b = 21.397(14) Å	b = 14.7766(4) Å
	c = 18.7960(3) Å	c = 12.039(8) Å	c = 21.1198(5) Å
	α = 90 °	α = 90 °	α = 90 °
	β = 94.1710(18) °	β = 93.162(8) °	β = 104.487(2) °
	γ = 90 °	γ = 90 °	γ = 90 °
Volume	3701.06(12) Å^3	3021(4) Å^3	3856.19(17) Å^3
Z	4	2	2
Density (calculated)	1.740 Mg·m^{-3}	1.934 Mg·m^{-3}	1.782 Mg·m^{-3}
Absorption coefficient	4.840 mm^{-1}	5.919 mm^{-1}	4.656 mm^{-1}
F_{000}	1920	1728	2064
Crystal size	0.201 · 0.108 · 0.047 mm^3 (plate)	0.300 · 0.100 · 0.060 mm^3 (block)	0.152 · 0.058 · 0.027 mm^3 (irregular)
θ range for data collection	2.173 to 34.153 °	1.903 to 30.508 °	2.422 to 28.312 °
Reflections collected	211338	69868	66576
Independent reflections [R_{int}]	14933 [0.1102]	9229 [0.0543]	9569 [0.0851]
Observed reflections [$I > 2\sigma(I)$]	11073	7302	7384
Index ranges *h, k, l*	-22 ... 22, -21 ... 21, -29 ... 29	-16 ... 16, -30 ... 30, -17 ... 17	-17 ... 17, -19 ... 19, -28 ... 28
Completeness to θ = 25.242 °	100.00%	100.00%	99.90%
Absorption correction	Gaussian	Semi-empirical from equivalents	Gaussian
Transmission factors: max, min	1.000, 0.477	0.4949, 0.3100	1.000, 0.631
Structure solution	heavy[(a)]	iterative[(b)]	dual[(c)]
Refinement method[(d)]	Full-matrix least-squares on F^2	Full-matrix least-squares on F^2	Full-matrix least-squares on F^2
Data / restraints / parameters	14933 / 0 / 521	9229 / 0 / 451	9569 / 36 / 530
Goodness-of-fit on F^2	1.099	1.072	1.032
Final R indices [$F_o > 4\sigma(F_o)$] $R(F)$, $wR(F^2)$	0.0423, 0.0745	0.0290, 0.0617	0.0373, 0.0764
Final R indices (all data) $R(F)$, $wR(F^2)$	0.0729, 0.0820	0.0485, 0.0691	0.0583, 0.0837
Difference density: rms, max, min	0.171, 1.905, -1.931 e·Å^{-3}	0.144, 2.548, -0.971 e·Å^{-3}	0.138, 2.126, -1.114 e·Å^{-3}
Diffractometer	Agilent SuperNova	Bruker AXS Smart 1000	Agilent SuperNova

[(a)] DIRDIF (Beurskens et al., 1999-2008); [(b)] SUPERFLIP (Palatinus, 2007-2009); [(c)] SIR2014 (Giacovazzo et al., 2014); [(d)] ShelXL (Sheldrick, 2015) Vers. 2018/3.

	CCDC 1916441	CCDC 1916442
Identification code	**co_mkn29_sq**	**co_mkn33**
Empirical formula	**$[Bi^{III}(L^1)(NO_3)](NO_3)$**·MeOH	**$[Bi^{III}(L^3)(Br)_{0.6}(NO_3)_{0.4}]$**·MeOH
	$C_{35}H_{37}BiN_8O_{14}$	$C_{31}H_{33}BiBr_{0.58}N_{5.42}O_{11.27}$
Formula weight	1002.7	916.91
Temperature	120(1) K	100(1) K
Wavelength	Mo K_α, 0.71073 Å	Mo K_α, 0.71073 Å
Crystal system	monoclinic	monoclinic
Space group	$P\ 2_1/n$ (*IT* Nr. 14)	$I\ a$ (*IT* Nr. 9)
Space group (Hall symbol)	-P 2yn	I -2ya
Unit cell dimensions	a = 10.53590(14) Å	a = 17.317(8) Å
	b = 22.8624(3) Å	b = 10.881(5) Å
	c = 16.7338(2) Å	c = 17.923(8) Å
	α = 90 °	α = 90 °
	β = 101.9182(15) °	β = 108.329(9) °
	γ = 90 °	γ = 90 °
Volume	3943.89(10) $Å^3$	3206(2) $Å^3$
Z	4	4
Density (calculated)	1.689 $Mg{\cdot}m^{-3}$	1.900 $Mg{\cdot}m^{-3}$
Absorption coefficient	4.548 mm^{-1}	6.292 mm^{-1}
F_{000}	1992	1801
Crystal size	0.199 · 0.027 · 0.022 mm^3 (needle)	0.270 · 0.200 · 0.100 mm^3 (irregular)
θ range for data collection	2.287 to 30.508 °	2.222 to 32.420 °
Reflections collected	88627	38878
Independent reflections [R_{int}]	12024 [0.0699]	10333 [0.0294]
Observed reflections [$I > 2\sigma(I)$]	9802	9773
Index ranges h, k, l	-15 ... 15, -32 ... 32, -23 ... 23	-26 ... 25, -16 ... 16, -26 ... 26
Completeness to θ = 25.242 °	99.90%	100.00%
Absorption correction	Gaussian	Semi-empirical from equivalents
Transmission factors: max, min	1.000, 0.442	0.3391, 0.2070
Structure solution	iterative[(a)]	dual[(b)]
Refinement method[(c)]	Full-matrix least-squares on F^2	Full-matrix least-squares on F^2
Data / restraints / parameters	12024 / 0 / 528	10333 / 2 / 473
Goodness-of-fit on F^2	1.074	0.762
Final R indices [$F_o > 4\sigma(F_o)$] $R(F)$, $wR(F^2)$	0.0335, 0.0628	0.0148, 0.0325
Final R indices (all data) $R(F)$, $wR(F^2)$	0.0481, 0.0667	0.0165, 0.0329
Difference density: rms, max, min	0.127, 2.869, -1.077 $e{\cdot}Å^{-3}$	0.075, 1.447, -0.478 $e{\cdot}Å^{-3}$
Diffractometer	Agilent SuperNova	Bruker AXS Smart 1000

[(a)] SUPERFLIP (Palatinus, 2007-2009); [(b)] SHELXT (Sheldrick, 2013); [(c)] ShelXL (Sheldrick, 2015) Vers. 2018/3.

4. Radiolabeling

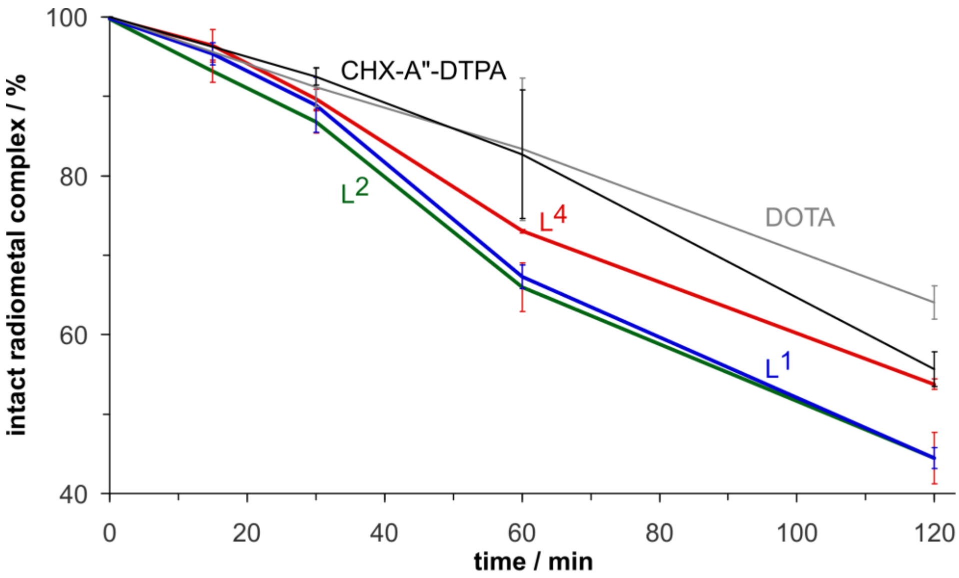

Figure S21. Percentage of intact $^{213}Bi^{III}$ complexes as functions of time upon challenge with 0.1 M aqueous sodium DTPA (pH 7.5, 37 °C). Labeling prior to challenge was done at pH 5 for 5 min at 95 °C; data are given as mean values ± SD, *n* = 3. Data for CHX-A''-DTPA and DOTA are taken from the literature[31] and shown for comparison.

5. Computational Work

The general method for the computation of log $D_{7.4}$ values based on the fluctuating charge model[32, 33] was extended to also be applicable for Bi^{III}, In^{III}, La^{III} and Lu^{III}. In contrast to the previous publications,[32, 33] the reference QM wave function for the parameterization of the fluctuating charge model was exclusively derived from crystallographic data via single point calculations with the DZP basis set,[34, 35] followed by a wavefunction analysis with the program Chargemol,[36, 37] to obtain DDEC6[37] monopole charges, from which the charge-derived descriptors were calculated. The force field atom types for the reference and test complexes were obtained in an automatic fashion. For the QM-derived atomic charge *vs*. calculated charge fit see Figure S22.

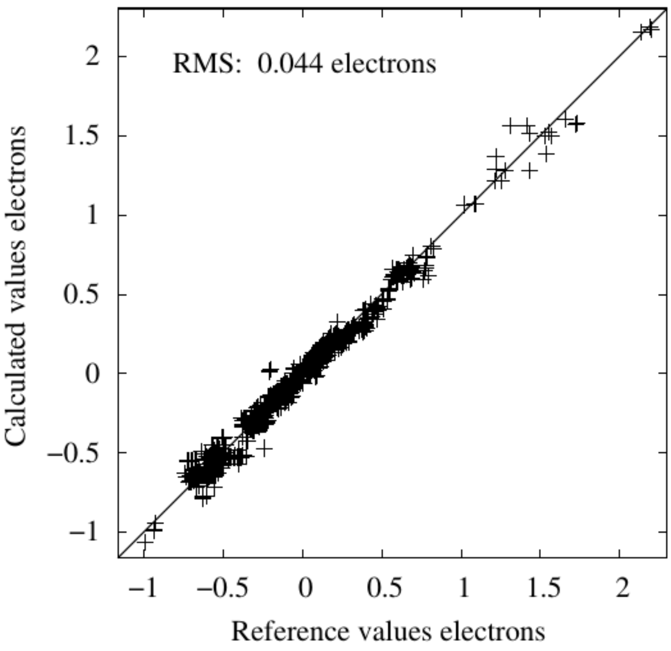

Figure S22. Atomic charges determined with the MOMEC fluctuating charge model *vs.* QM-derived atomic charges for the charge-parameterization dataset.

For the fit data set for the log $D_{7.4}$ values the MOMEC-optimized structures were used.[33] In addition, to be able to reproduce the experimental log $D_{7.4}$ values of the two Cu^{II} complexes with L^5 and L^6, which differ by more than one unit despite very similar structures, these were added to the fit data set. The effect on the predictions can be seen when comparing the isomeric complexes with L^5 and L^6, where the difference in log $D_{7.4}$ is more or less reproduced, with the set of isomeric Bi^{III} complexes with L^1 and L^2, where the predicted difference is qualitatively correct but much too small. The structures of the Bi^{III} and Cu^{II} complexes of L^1-L^9 were obtained from crystal structural data; where appropriate co-ligands were manually added or replaced.

The geometry-dependent descriptors used for the multiple regression fit to obtain log $D_{7.4}$ values were corrected with respect to the published procedure and values in the case of the ovality descriptor; moreover, the Glen polarizability descriptor[38] was added. The descriptors are: the solvent-accessible surface area (SAS), the ovality, the polarity and the total variance. In addition, binned surface potentials for the electric potential (*ebp*) and for the glen polarizability (*pbp*) were used; the surface area for the given range (*e.g.* for the pbp_2.0 descriptor, the [2.0-2.5] interval) were only added to the descriptor value if the surface patch

constructed from the respective interval of the mapped surface property exceeded an area of 1.0 Å^2.

Given the limited number of experimental data we limited the number of parameters to 13, avoiding the problem of over-parameterization. Note that the Glen polarizability is based on Slater's rules constants and therefore on our atomic charge scheme, so that except for the solvent accessible surface area and the ovality, all parameters directly or indirectly depend on the MOMEC-computed atomic charges.

Two fits are presented below, where the second includes both the experimental data from the previous QSPR study as well as the experimental data presented here, which overall leads to a better accuracy in reproducing the experimental data.

The 13-parameter multiple regression fit obtained (R^2 (COD)=0.98, adj. R^2=0.97, 21 degrees of freedom remaining), only using the original training set, is given in eq. 1. The calculated *vs*. experimental values for the log $D_{7.4}$ data set is visualized in Figure S23.

$\log D_{7.4}$ = -73.23114 + area * -0.0414 + ebp_2.000 * -0.00915 + pbp_0.100 * 0.01991 + pbp_0.150 * 0.03418 + pbp_0.200 * 0.03085 + pbp_0.250 * 0.03704 + pbp_0.300 * 0.04166 + pbp_0.350 * 0.01076 + pbp_0.400 * 0.03644 + pbp_0.500 * -0.02858 + ovality * 75.0373 + polarity * 5.8028 + variance * -2.9593; Eq. 1

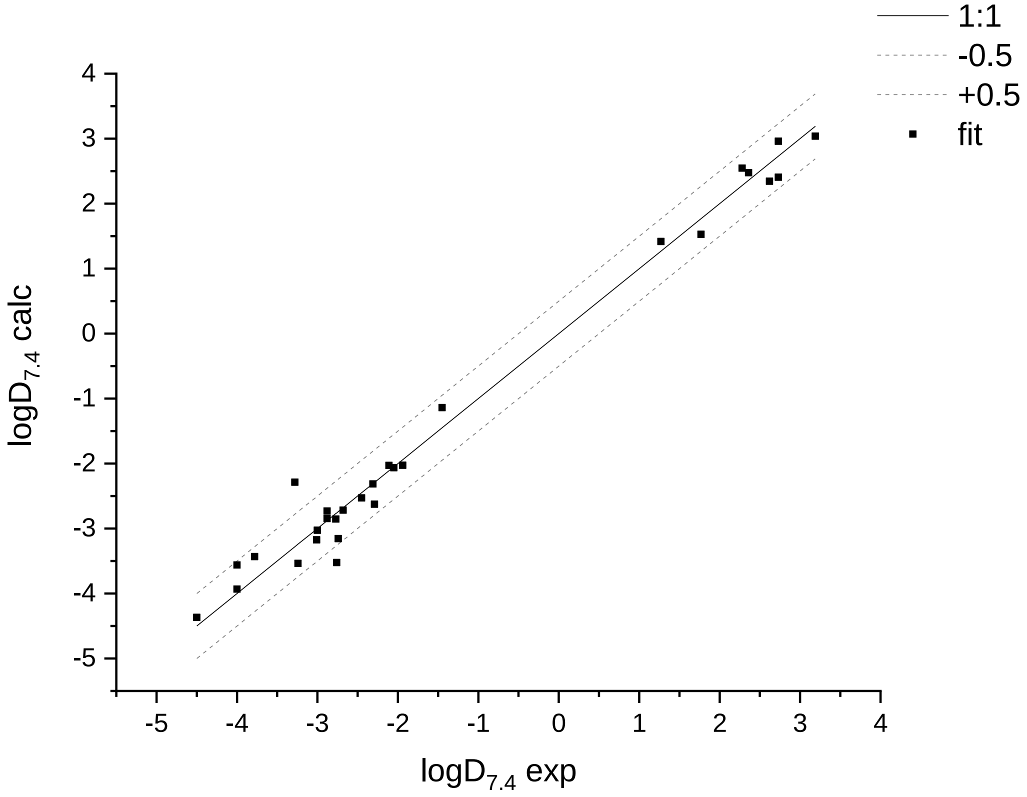

Figure S23. Multiple linear regression log $D_{7.4}$ fit of 29 structures from [32, 33] with the regression formula eq.1 (R^2=0.978, adj. R^2=0.965).

The 13-parameter multiple regression fit obtained (R^2 (COD)=0.98, adj. R^2=0.96, 35 degrees of freedom remaining), also including the parameters from this study is given in eq. 2. The calculated *vs*. experimental values for the log $D_{7.4}$ data set is visualized in Figure S24.

$\log D_{7.4}$ = -68.36704 + area * -0.04294 + ebp_2.000 * -0.01084 + ovality * 68.54903 + pbp_0.100 * 0.02367 + pbp_0.150 * 0.04217 + pbp_0.200 * 0.0348 + pbp_0.250 * 0.0466 + pbp_0.300 * 0.04278 + pbp_0.350 * 0.02297 + pbp_0.400 * 0.03424 + pbp_0.500 * -0.01929 + polarity * 5.22349 + variance * -2.54263; Eq. 2

.

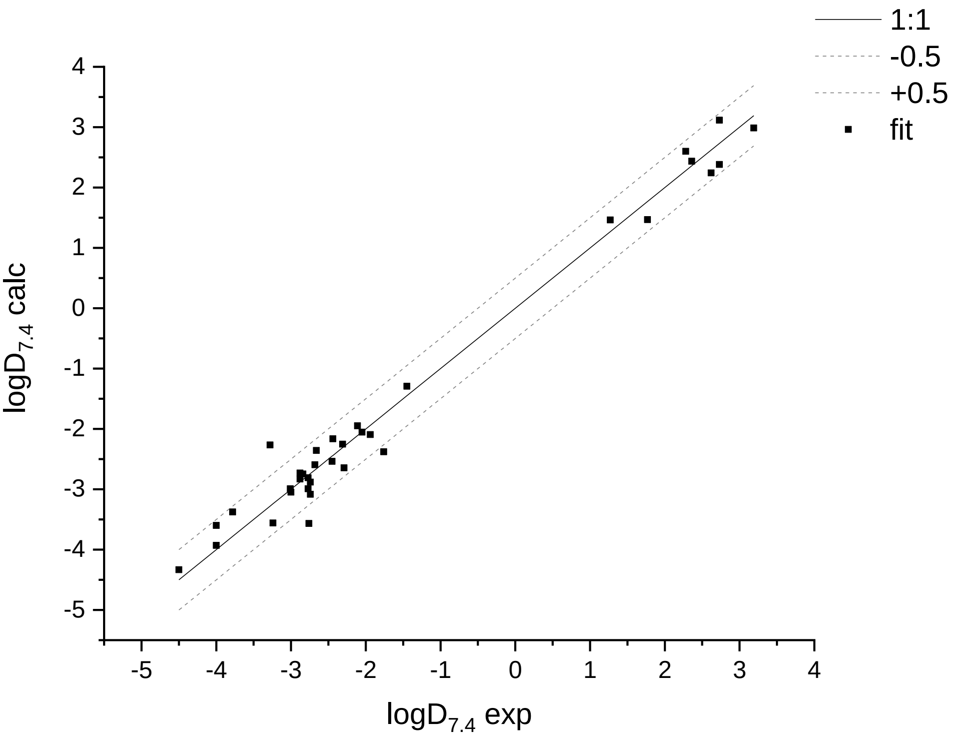

Figure S24. Multiple linear regression log $D_{7.4}$ fit of 36 structures from [32, 33] and from L^1-L^2,L^4-L^9 with the regression formula eq.2 (R^2=0.979, adj. R^2=0.966).

Note that the present fits are based on a limited data set and a limited set of parameters. There certainly is the possibility to further extend the scope of the approach, especially with a larger data set and also including different metal ions and further optimize the current fitting procedure.

The large variation of the predicted values in dependence of the co-ligand will need further refinement. The problem arises with complexes that are not coordinatively saturated and where a solvent molecule or counter anion complete the coordination sphere (note that this is also the case for some complexes used in the training set). The question then is, whether or not a coordinated anion is replaced by a solvent molecule. Because this depends on the bond strength of the metal ion to the monodentate ligand and on the solvent, the coordination sphere might even differ in H_2O and octanol, and therefore further complicating the situation. The other problem of course is that the overall structure of the complex might change significantly between condensed and solution phase. It appears that modeling (MM or DFT)

of the structures might be preferable from using crystal structures but this is a question that needs to be studied in more detail. Table S2 shows the results of the above fits applied to the complexes of L^1-L^9 for the first fit (eq. 1), and Table S3 lists the corresponding data for the second fit (eq. 2).

The correspondence between experimental and predicted values is satisfactory. For the Bi^{III} complexes with different co-ligands the log $D_{7.4}$ values have been computed for no co-ligand, NO_3^-, H_2O and OH^-. For the Cu^{II}-L^8 complex both the dihydroxy (-2.72/-2.74) and keto (-2.59/-2.55) forms have been computed, with a moderate effect on the log $D_{7.4}$ values, as expected.

The orientation of the methoxy groups of the bispidine complexes (ester substituents at C1 and C5) can have a large effect on the predicted log $D_{7.4}$ values (rotation of the ester groups of the bispidine platform). In a calculation starting from complexes with the methoxy groups pointing approximately in the same direction (not shown here), the log $D_{7.4}$ value for the Cu^{II}-L^5 complex is -3.06, when one methoxy group is rotated to match the Cu^{II}-L^6 complex solid state structure, it is -3.63 (very close to the experimental value of -3.78).

Table S2. Experimental and computed log $D_{7.4}$ values of Bi^{III} and Cu^{II} bispidine complexes (eq.1)

complex		*experimental*	*computed, co-ligand*					
			--	NO_3^-	H_2O	OH^-	C^9-keto	Cl^-
$^{213}Bi^{III}$	L^1	-1.76	-2.68	-2.68	-3.07	-3.43	--	--
	L^2	-2.66	-2.76	--	-3.14	-3.44	--	--
	L^3	--	-3.76	-1.78	-4.04	-1.59	--	--
	L^4	-2.74	-3.67	-3.16	-3.84	-2.89	--	--
$^{64}Cu^{II}$	L^5	-3.78	-3.41	--	--	--	--	--
	L^6	-2.74	-3.15	--	--	--	--	--
	L^7	-2.77	-2.84	--	--	--	--	--
	L^8	-2.84	-2.72	--	--	--	-2.59	--
	L^9	-2.44	-2.21	--	-2.29	--	--	-8.44

Table S3. Experimental and computed log $D_{7.4}$ values of Bi^{III} and Cu^{II} bispidine complexes (eq.2)

complex		*experimental*	*computed, co-ligand*					
			--	NO_3^-	H_2O	OH^-	C^9-keto	Cl^-
$^{213}Bi^{III}$	L^1	-1.76	-2.35	-3.48	-2.58	-3.04	--	--
	L^2	-2.66	-2.35	--	-2.61	-2.98	--	--
	L^3	--	-3.43	-1.31	-3.54	-1.31	--	--
	L^4	-2.74	-3.09	-2.53	-3.20	-2.32	--	--
$^{64}Cu^{II}$	L^5	-3.78	-3.36	--	--	--	--	--
	L^6	-2.74	-2.87	--	--	--	--	--
	L^7	-2.77	-2.80	--	--	--	--	--
	L^8	-2.84	-2.74	--	--	--	-2.55	--
	L^9	-2.44	-2.16	--	-2.22	--	--	-8.42

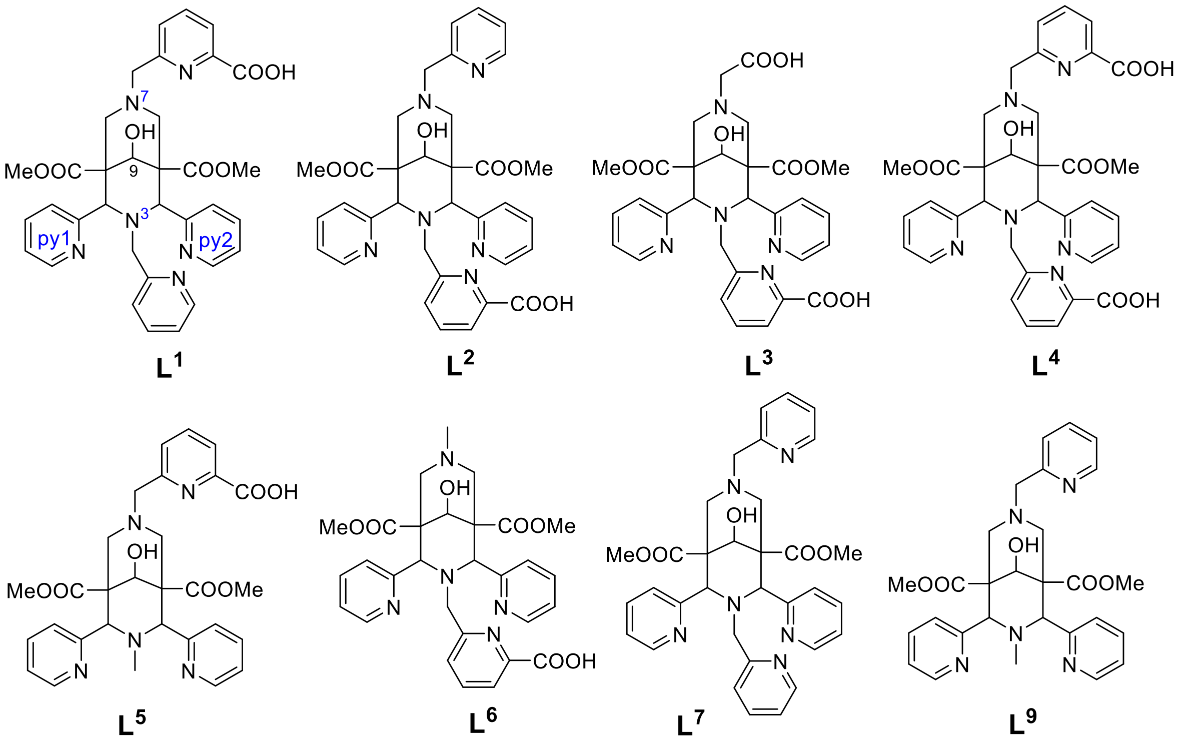

L^8 is a derivative of L^7 with a C9 ketone (instead of the alcohol)

The MOMEC program, also including the modules for the calculation of the charge distribution and the log $D_{7.4}$ values, is regularly improved. The current version is available free of charge from the authors (bodo.martin@aci.uni-heidelberg.de).

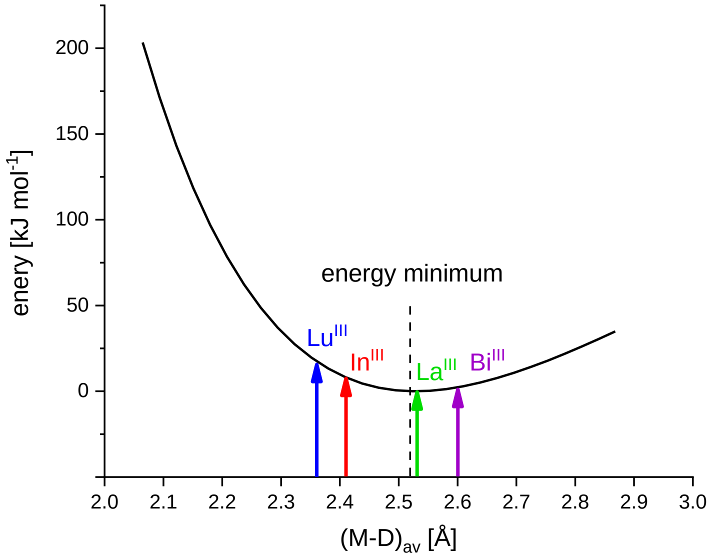

Figure S25. Hole size (and shape) curve for L^4 (adopted from [1]). The strain energy (MOMEC program and force field[41-44]) is plotted as a function of the averaged metal-donor distances $(M\text{-}D)_{av}$ with the minimum of the curve set to 0 kJ mol^{-1}. Apart from the metal-donor atom-ligand backbone angle deformation, the curve does not include any metal-ion-dependent terms. The variation of the metal-donor atom distances was asymmetric (i.e., the shape of the ligand and its variation were taken into account). The approximation adopted included full geometry optimization of the Zn^{II} complex as a large metal ion and the Co^{III} complex as a small metal ion and linear approximation between these structures to determine the relative changes in metal-donor atom distance for all eight bonds.

References

[1] P. Comba, U. Jermilova, C. Orvig, B. O. Patrick, C. F. Ramogida, K. Rück, C. Schneider, M. Starke, *Chem. Eur. J.* **2017**, *23*, 15945-15956.

[2] K. Kabsch, in *International Tables for Crystallography, Vol. F, Ch.11.3* (Ed.: M. G. Rossmann, Arnold, E.), Kluwer Academic Publishers, Dordrecht, **2001**.

[3] Bruker, 1997-2019, SAINT, Bruker AXS GmbH, Karlsruhe, Germany

[4] CrysAlisPro, 2011-2014, CrysAlisPro, Oxford, Agilent Technologies, UK Ltd.

[5] R. O. Diffraction, 2015-2019, Rigaku Oxford Diffraction, Wroclaw, Poland, Rigaku Polska Sp.zo.o.

[6] R. H. Blessing, *Acta Cryst.* **1995**, *A51*, 33-38.

[7] G. M. Sheldrick, 2004-2014, *SADABS*, Karlsruhe, Germany, Bruker AXS GmbH

[8] L. Krause, R. Herbst-Irmer, G. M. Sheldrick, D. Stalke, *J. Appl. Cryst.* **2015**, *48*, 3-10.

[9] CrysAlisPro, 2011-2014, SCALE3 ABSPACK CrysAlisPro, Oxford, Agilent Technologies UK Ltd.

[10] W. R. Busing, H. A. Levy, *Acta Cryst.* **1957**, *10*, 180-182.

[11] G. M. Sheldrick, 2012-2018, SHELXT, University of Göttingen and Bruker AXS GmbH, Karlsruhe, Germany

[12] M. Ruf, B. C. Noll, 2014, Application Note SC-XRD 503, Karlsruhe, Germany, Bruker AXS GmbH
[13] G. M. Sheldrick, *Acta Cryst.* **2015**, *C71*, 3-8.
[14] P. T. Beurskens, G. Beurskens, R. de Gelder, J. M. M. Smits, S. Garcia-Granda, R. O. Gould, 2008, DIRDIF-2008, DIRDIF-2008, Radboud University Nijmegen, The Netherlands
[15] P. T. Beurskens, in *Crystallographic Computing 3* (Eds.: G. M. Sheldrick, C. Krüger, R. Goddard), Clarendon Press, Oxford, UK, **1985**, p. 216.
[16] M. C. Burla, R. Caliandro, B. Carrozzini, G. L. Cascarano, C. Cuocci, C. Giacovazzo, M. Mallamo, A. Mazzone, G. Polidori, 2014, SIR2014, CNR IC, Bari, Italy,
[17] M. C. Burla, R. Caliandro, B. Carrozzini, G. L. Cascarano, C. Cuocci, C. Giacovazzo, M. Mallamo, A. Mazzone, G. Polidori, *J. Appl. Cryst.* **2015**, *48*, 306-309.
[18] L. Palatinus, 2007-2014, SUPERFLIP, EPF Lausanne, Switzerland and Fyzikalni ustav AV CR, Prague, Czech Republic
[19] L. Palatinus, G. Chapuis, *J. Appl. Cryst.* **2007**, *40*, 786-790.
[20] G. M. Sheldrick, 2012-2018, SHELXL-20xx,, University of Göttingen and Bruker AXS GmbH, Karlsruhe, Germany
[21] W. Robinson, G. M. Sheldrick, in *Crystallographic Computing 4* (Eds.: M. Isaacs, M. R. Taylor), Ch.22, IUCr and Oxford University Press, Oxford, UK, **1988**.
[22] G. M. Sheldrick, *Acta Cryst.* **2008**, *A64*, 112-122.
[23] J. S. Rollett, in *Crystallographic Computing* (Eds.: F. R. Ahmed, S. R. Hall, C. P. Huber), Munksgaard, Copenhagen, Denmark, **1970**, p. 167.
[24] D. Watkin, in *Crystallographics Computing 4* (Eds.: N. W. Isaaks, M. R. Taylor), Ch. 8, IUCr and Oxford University Press, Oxford, UK, **1988**.
[25] P. Müller, R. Herbst-Irmer, A. L. Spek, T. R. Schneider, M. R. Sawaya, in *Crystal Structure Refinement, Vol. Ch. 5* (Ed.: P. Müller), Oxford University Press, Oxford, **2006**.
[26] D. Watkin, *J. Appl. Cryst.* **2008**, *41*, 491.
[27] P. v. d. Sluis, A. L. Spek, *Acta Cryst.* **1990**, *A46*, 194-201.
[28] A. L. Spek, *Acta Cryst.* **2015**, *C71*, 9-18.
[29] A. L. Spek, 2003, PLATON, Utrecht University, The Netherlands
[30] A. L. Spek, *J. Appl. Cryst.* **2003**, *36*, 7-13.
[31] J. Šimeček, P. Hermann, C. Seidl, F. Bruchertseifer, A. Morgenstern, H.-J. Wester, J. Notni, *EJNMMI Research* **2018**, *8*, 78.
[32] P. Comba, B. Martin, A. Sanyal, *J. Comput. Chem.* **2013**, *34*, 1598-1608.
[33] P. Comba, B. Martin, A. Sanyal, H. Stephan, *Dalton Trans.* **2013**, *42*, 11066-11073.
[34] E. V. R. de Castro, *J. Chem. Phys.* **1998**, *108*, 5225.
[35] F. E. Jorge, L. S. C. Martins, M. L. Franco, *Chem. Phys. Lett.* **2016**, *643*, 84-88.
[36] T. A. Manz, D. S. Sholl, *J. Chem. Theory Comput.* **2010**, *6*, 2455-2468.
[37] T. A. Manz, N. Gabaldon Limas, *RSC Adv.* **2016**, *6*, 45727-45747.
[38] R. C. Glen, *J. Comp. Aided Mol. Design* **1994**, *8*, 457-466.
[39] S. Juran, M. Walther, H. Stephan, R. Bergmann, J. Steinbach, W. Kraus, F. Emmerling, P. Comba, *Bioconj. Chem.* **2009**, *20*, 347-359. DOI: 310.1021/bc800461e.
[40] P. Comba, S. Hunoldt, M. Morgen, J. Pietzsch, H. Stephan, H. Wadepohl, *Inorg. Chem.* **2013**, *52*, 8131-8143.
[41] P. Comba, T. W. Hambley, G. Lauer, N. Okon, *MOMEC97, a molecular modeling package for inorganic compounds*, Heidelberg, **1997**.
[42] P. V. Bernhardt, P. Comba, *Inorg. Chem.* **1992**, *31*, 2638.
[43] P. Comba, T. W. Hambley, M. Ströhle, *Helv. Chim. Acta.* **1995**, *78*, 2042-2047.
[44] J. E. Bol, C. Buning, P. Comba, J. Reedijk, M. Ströhle, *J. Comput. Chem.* **1998**, *19*, 512-523.

checkCIF/PLATON report

Structure factors have been supplied for datablock(s) co_mkn11, co_mkn17, co_mkn18, co_mkn21a, co_mkn24, co_mkn25_sq, co_mkn26a_sq, co_mkn29_sq, co_mkn33

THIS REPORT IS FOR GUIDANCE ONLY. IF USED AS PART OF A REVIEW PROCEDURE FOR PUBLICATION, IT SHOULD NOT REPLACE THE EXPERTISE OF AN EXPERIENCED CRYSTALLOGRAPHIC REFEREE.

No syntax errors found. CIF dictionary Interpreting this report

Datablock: co_mkn11

```
Bond precision:  C-C = 0.0015 A          Wavelength=0.71073

Cell:            a=11.46814(17)   b=16.70278(19)     c=13.9810(2)
                 alpha=90         beta=110.9754(16)  gamma=90
Temperature:     120 K

                Calculated                 Reported
Volume          2500.59(6)                 2500.60(6)
Space group     P 21/n                     P 21/n
Hall group      -P 2yn                     -P 2yn
Moiety formula  C27 H27 N5 O5, H2 O        C27 H27 N5 O5, H2 O
Sum formula     C27 H29 N5 O6              C27 H29 N5 O6
Mr              519.55                     519.55
Dx,g cm-3       1.380                      1.380
Z               4                          4
Mu (mm-1)       0.099                      0.099
F000            1096.0                     1096.0
F000'           1096.52
h,k,lmax        17,25,21                   17,25,21
Nref            8995                       8760
Tmin,Tmax       0.977,0.988                0.983,0.990
Tmin'           0.977

Correction method= # Reported T Limits: Tmin=0.983 Tmax=0.990
AbsCorr = GAUSSIAN

Data completeness= 0.974          Theta(max)= 32.419

R(reflections)= 0.0418( 7464)     wR2(reflections)= 0.1082( 8760)

S = 1.040                  Npar= 357
```
